# Supplementary material for: P720R USP8 Mutation Is Associated with a Better Responsiveness to Pasireotide in ACTH-Secreting PitNETs
Source: Cancers (Basel). 2022 May 16;14(10):2455. doi: 10.3390/cancers14102455 (PMC9139692; doi:10.3390/cancers14102455)
Supplement: Supplementary file 1 [file cancers-14-02455-s001.zip › cancers-1670385-supplementary.pdf]

Fig. S1 a)

FLAG-USP8 molecular weight

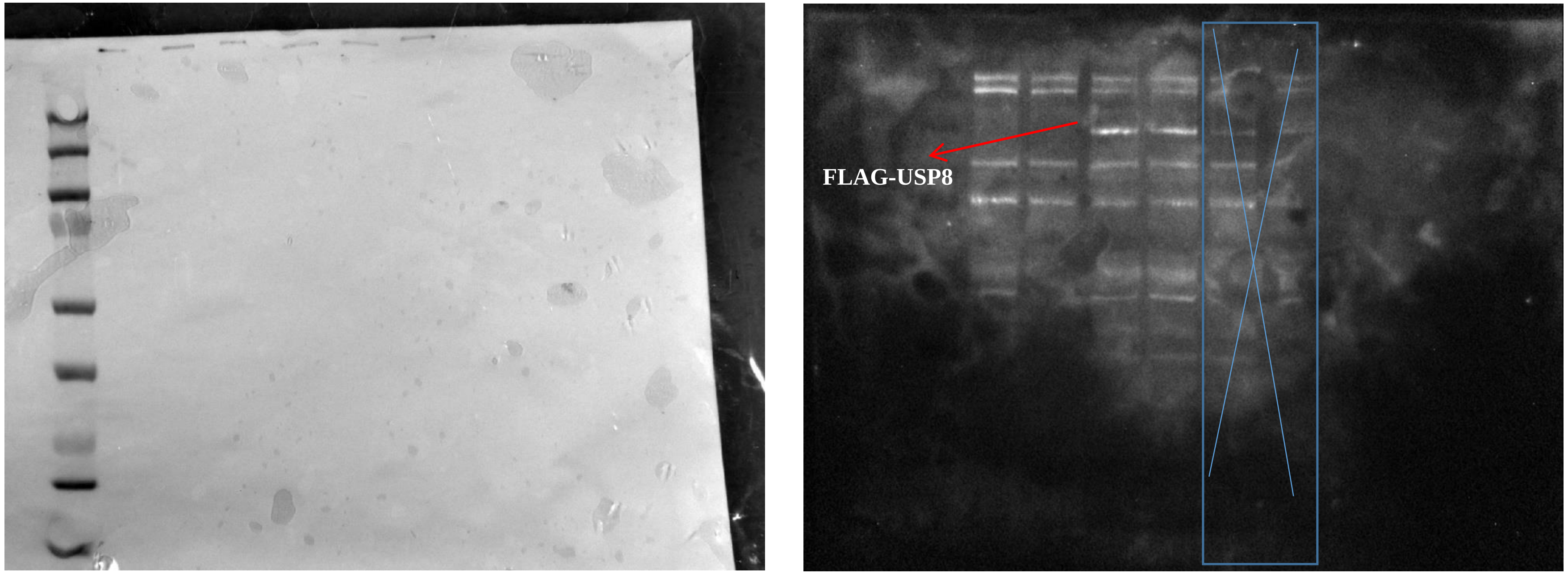

Fig. S1 a-b: Original western blots and merge with molecular weigth. Immunoblot of USP8 in USP8 wild-type ACTH-secreting Pit-NET primary cultures transfected with USP8-718del mutant and treated with pasiretoide. Membranes were incubated with USP8 (a) and GAPDH (b) antibodies.

b)

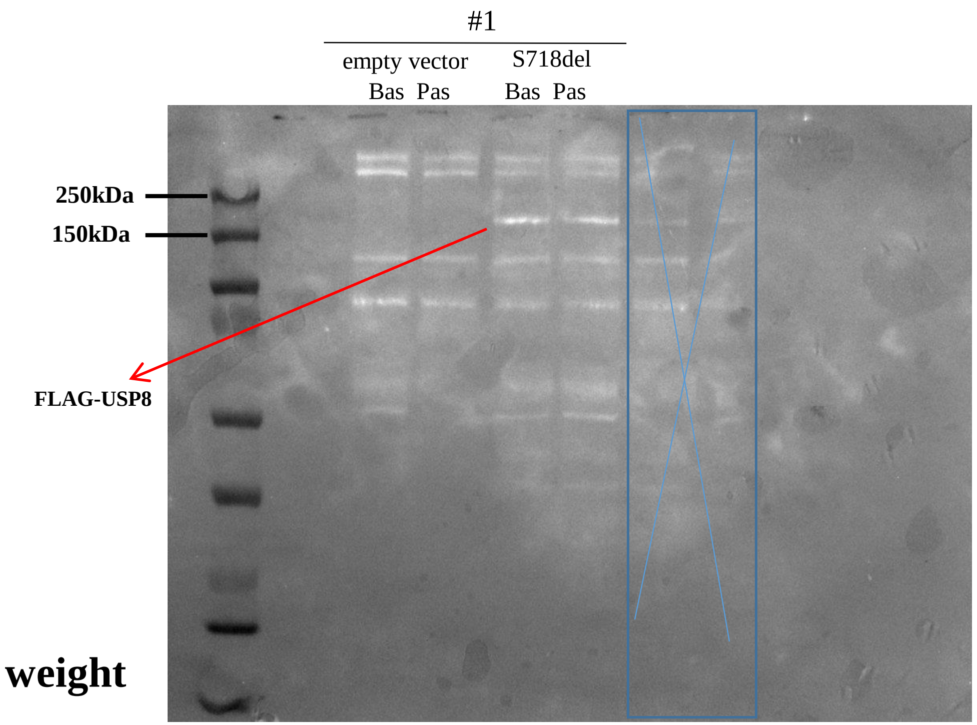

Merged with molecular weight

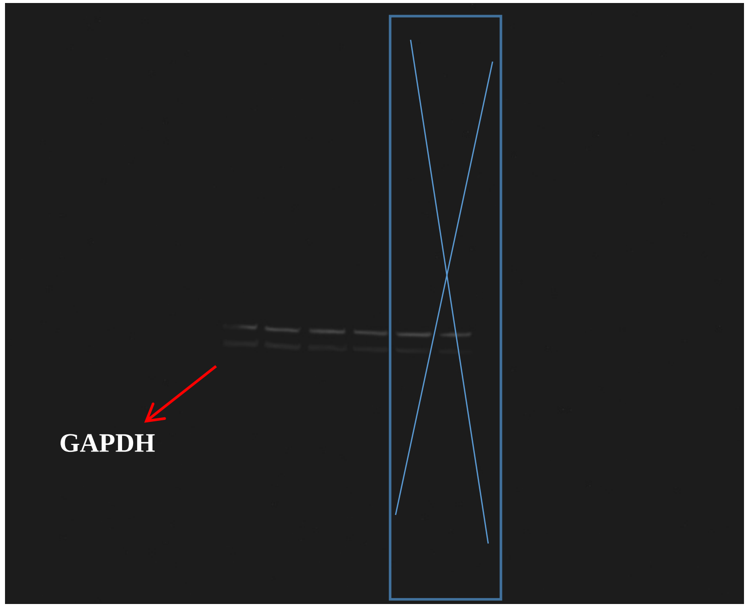

Fig. S1 c

molecular weight

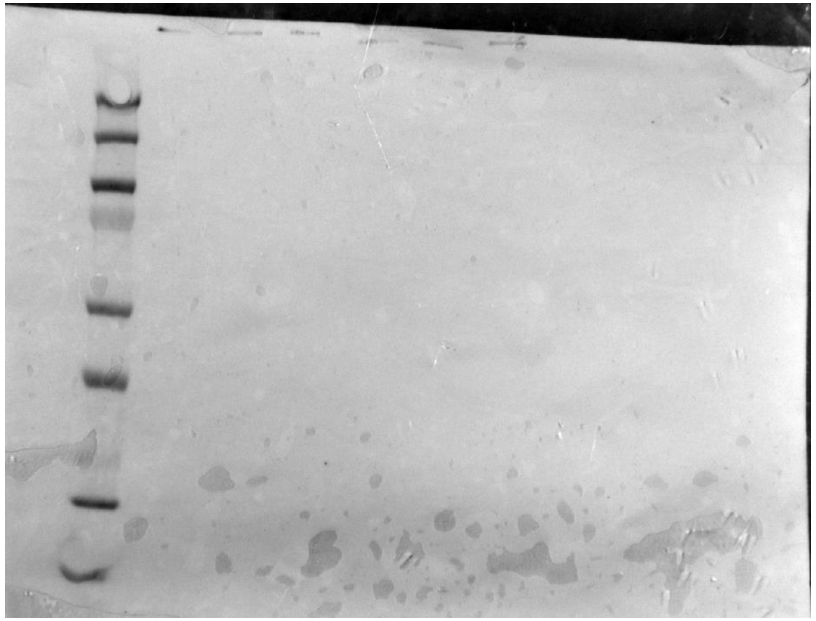

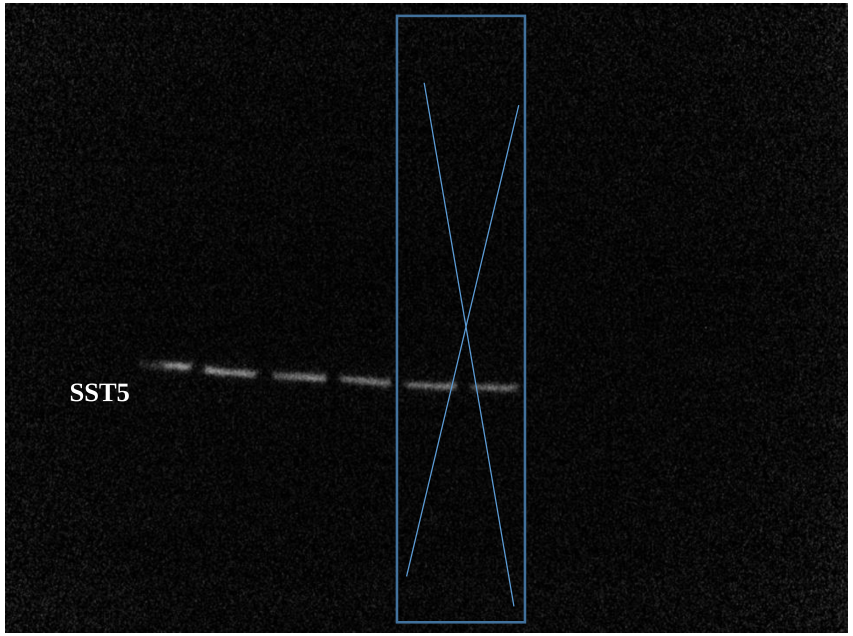

Merged with molecular weight

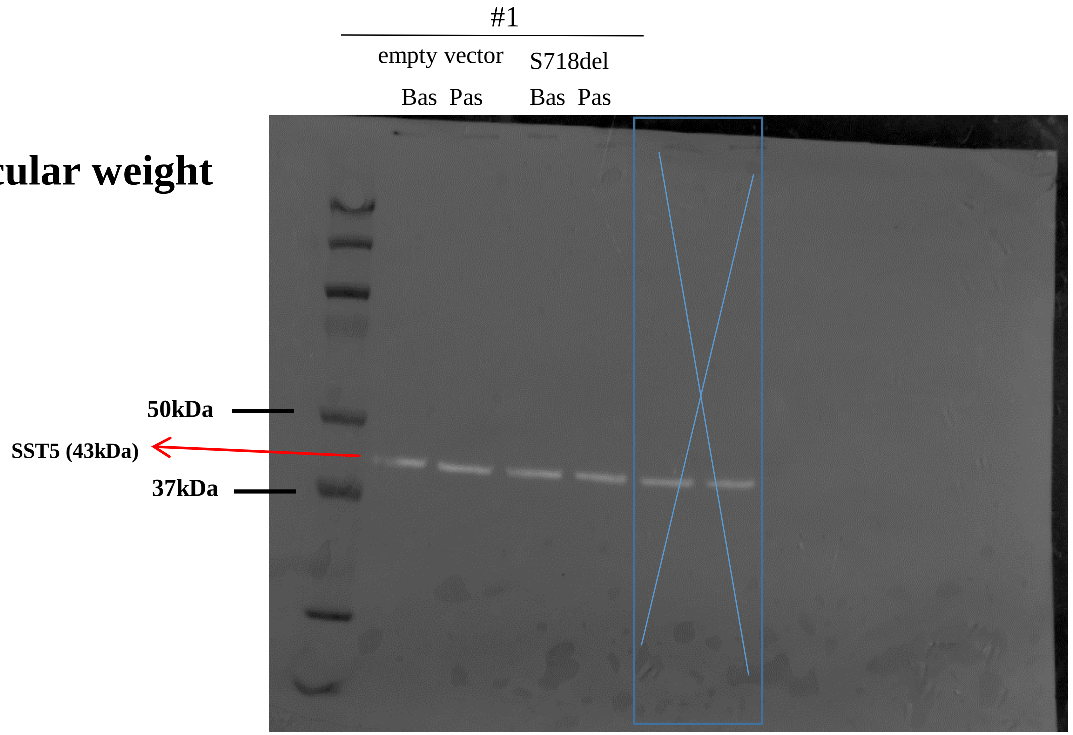

Fig. S1 c: Original western blots and merge with molecular weigth. Immunoblot of SST5 in USP8 wild-type ACTH-secreting Pit-NET primary cultures transfected with USP8-718del mutant and treated with pasiretoide. Membranes were incubated with SST5 antibody.

Fig. S1 d

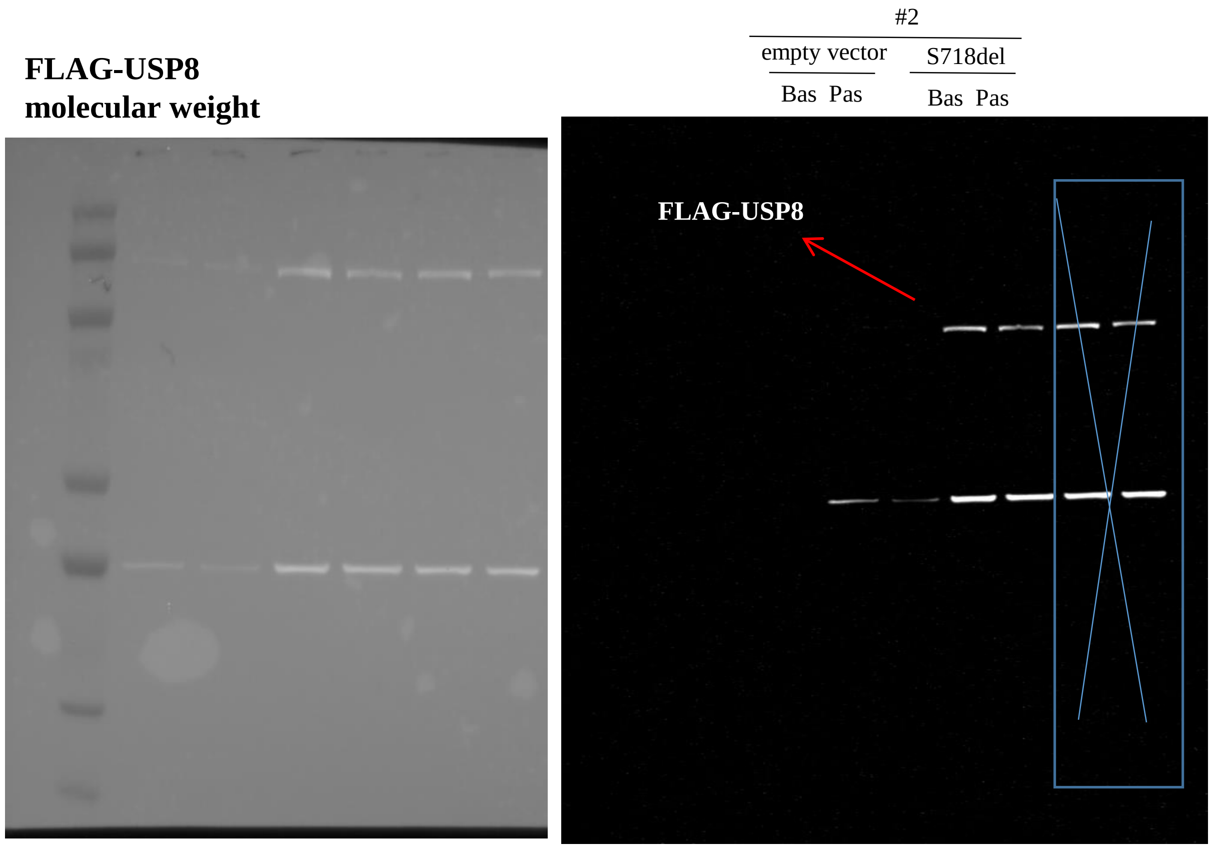

Merge with molecular weight

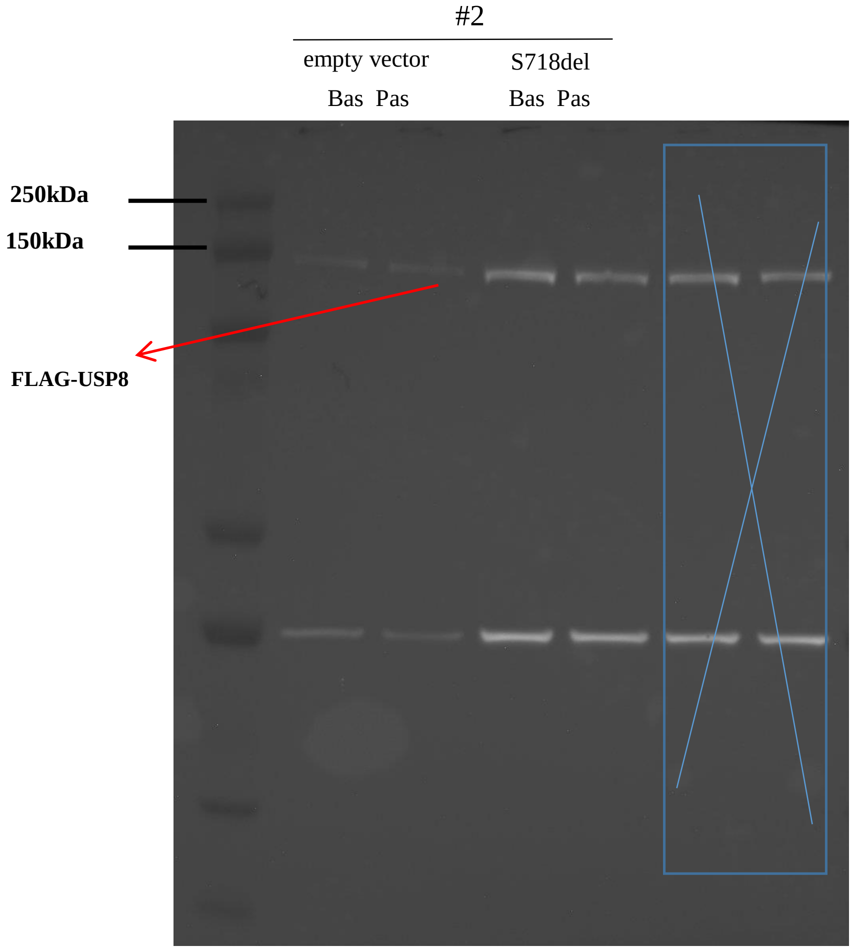

Fig. S1 d: Original western blots and merge with molecular weigth. Immunoblot of USP8 in USP8 wild-type ACTH-secreting Pit-NET primary cultures transfected with USP8-718del mutant and treated with pasiretoide. Membranes were incubated with USP8 antibody.

Fig. S1e

GAPDH
molecular weight

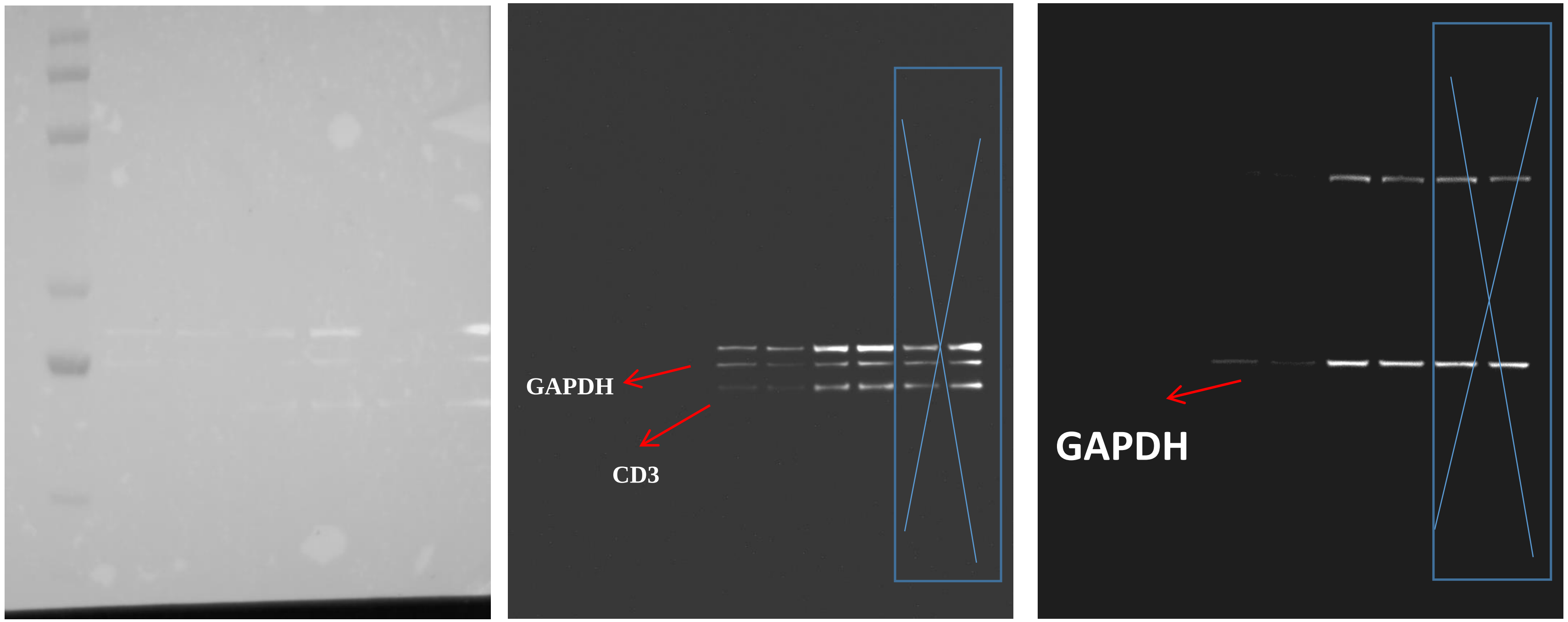

Merge with molecular weight

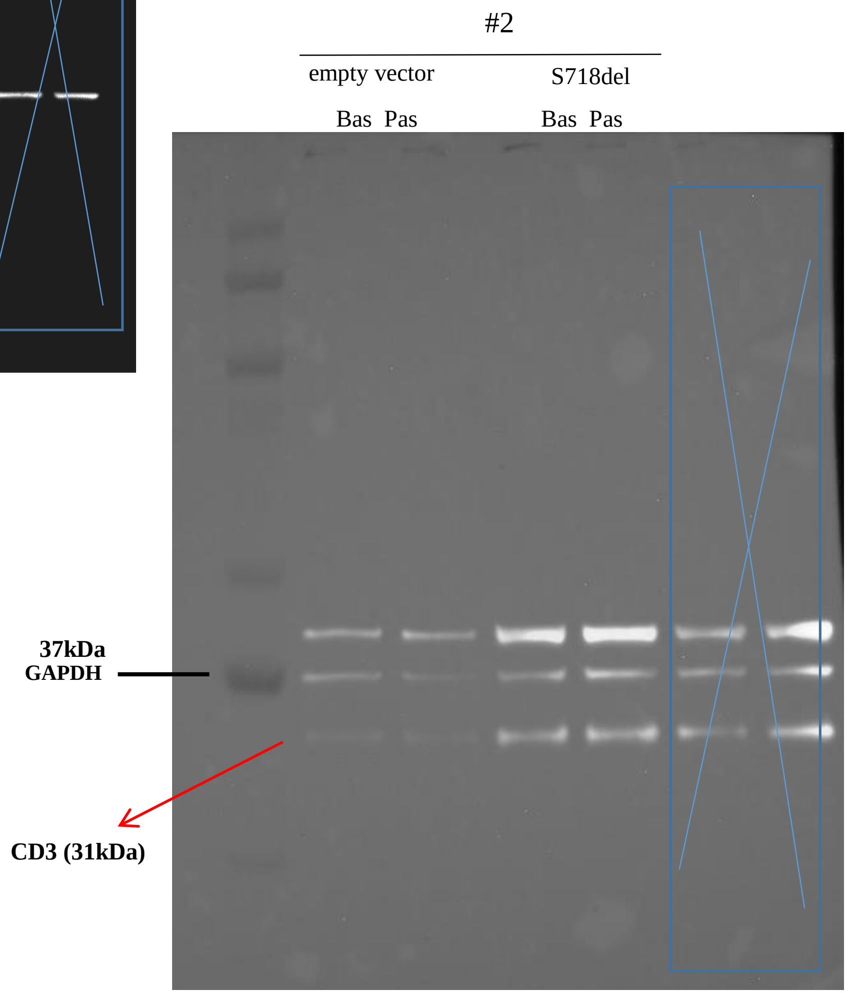

Fig. S1e: Original western blots and merge with molecular weight. Immunoblot of GAPDH in USP8 wild-type ACTH-secreting Pit-NET primary cultures transfected with USP8-718del mutant and treated with pasiretoide. Membranes were incubated with GAPDH antibody.

Fig. S1 f

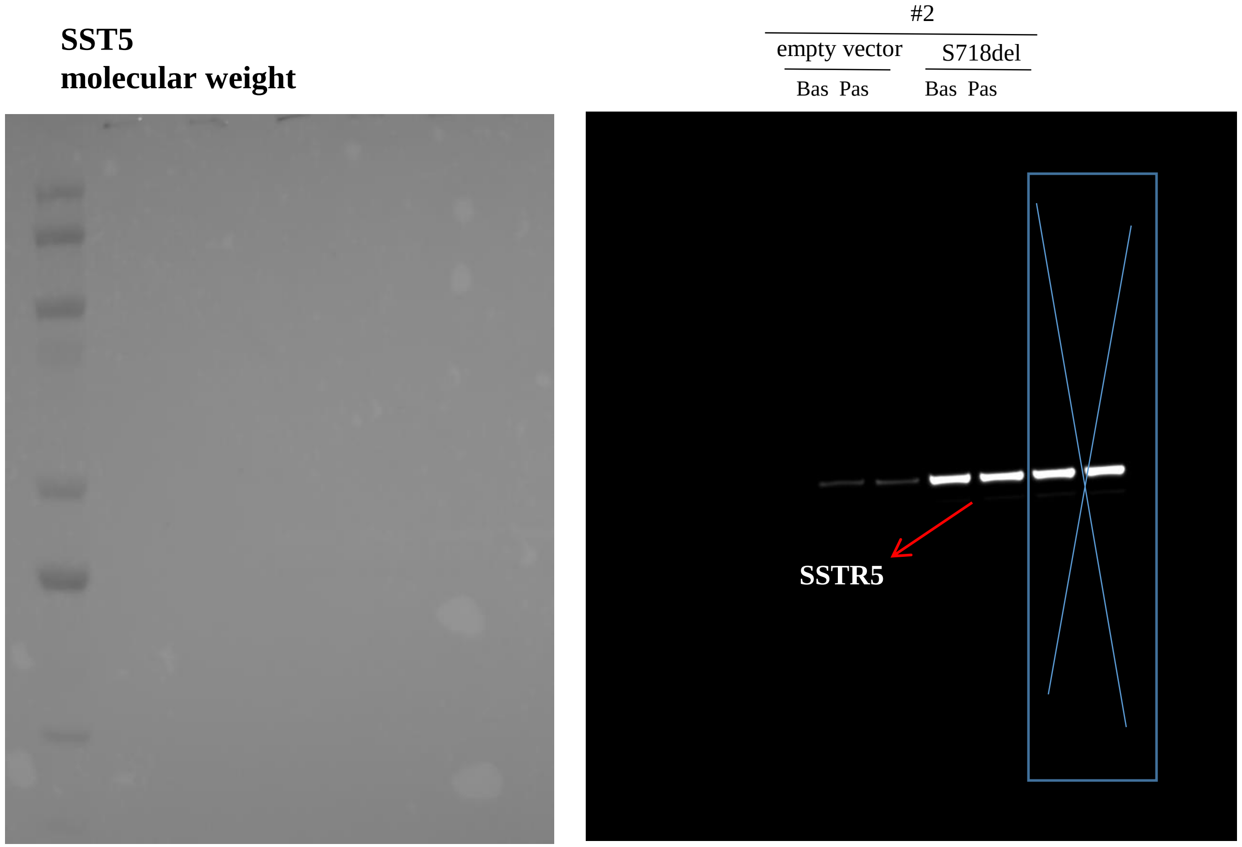

Merge with molecular weight

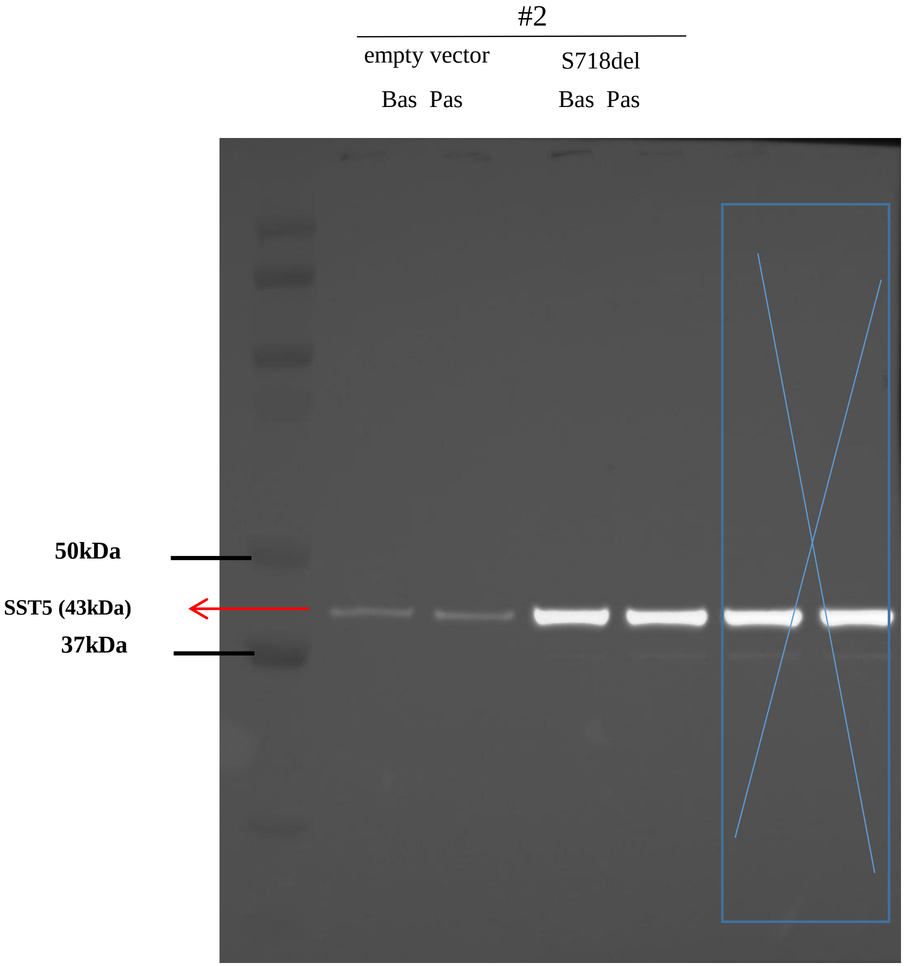

Fig. S1 f: Original western blots and merge with molecular weight. Immunoblot of SST5 in USP8 wild-type ACTH-secreting Pit-NET primary cultures transfected with USP8-718del mutant and treated with pasireotide. Membranes were incubated with SST5 antibody.

Fig. S1 g

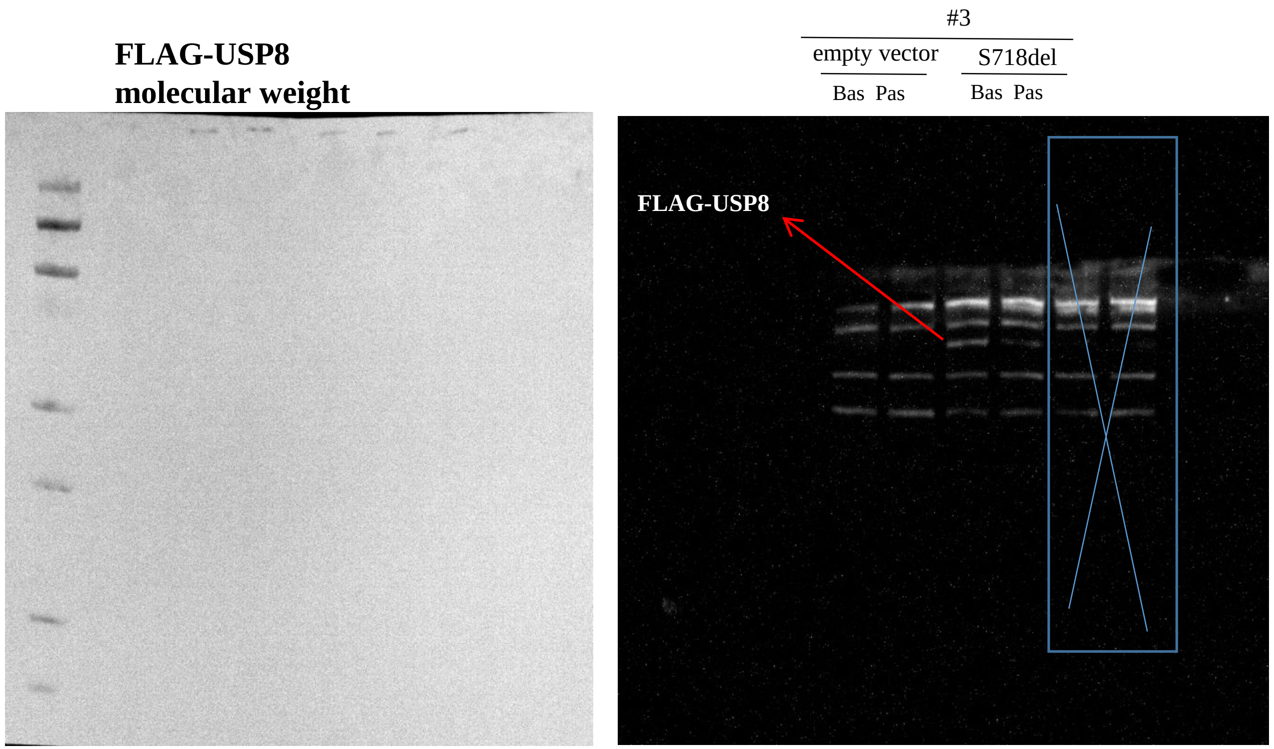

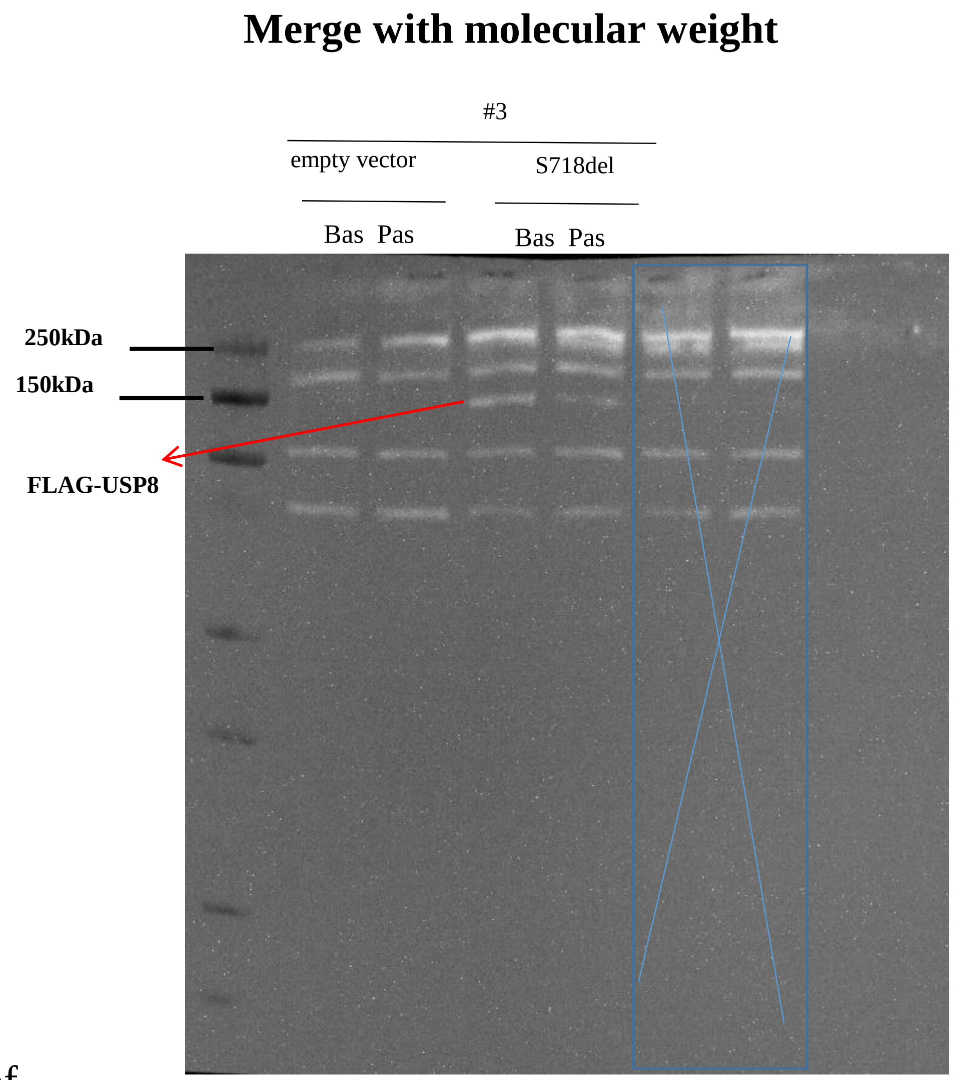

Fig. S1 g: Original western blots and merge with molecular weight. Immunoblot of USP8 in USP8 wild-type ACTH-secreting Pit-NET primary cultures transfected with USP8-718del mutant and treated with pasireotide. Membranes were incubated with USP8 antibody**.**

Fig. S1 h

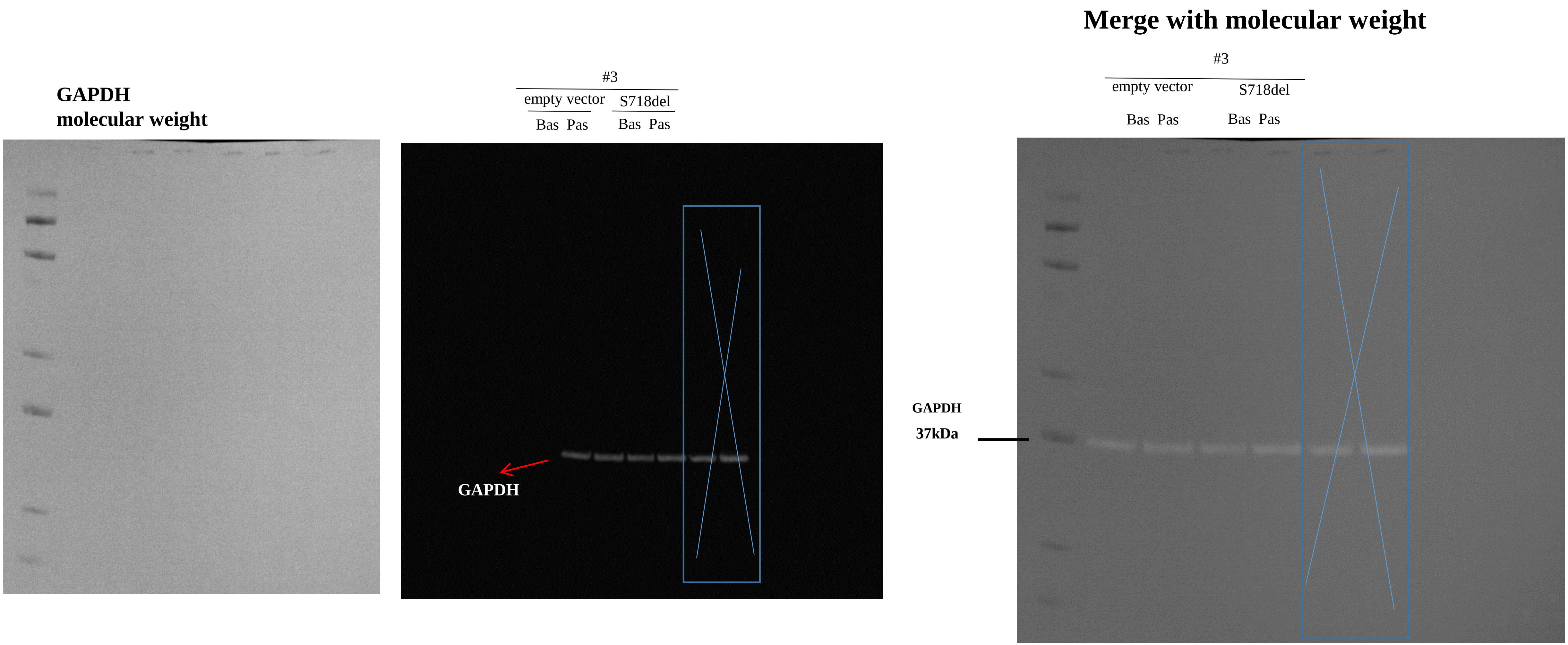

Fig. S1 h: Original western blot and merge with molecular weight. Immunoblot of GAPDH in USP8 wild-type ACTH-secreting Pit-NET primary cultures transfected with USP8-718del mutant and treated with pasireotide. Membranes were incubated with GAPDH antibody.

Fig. S1 i

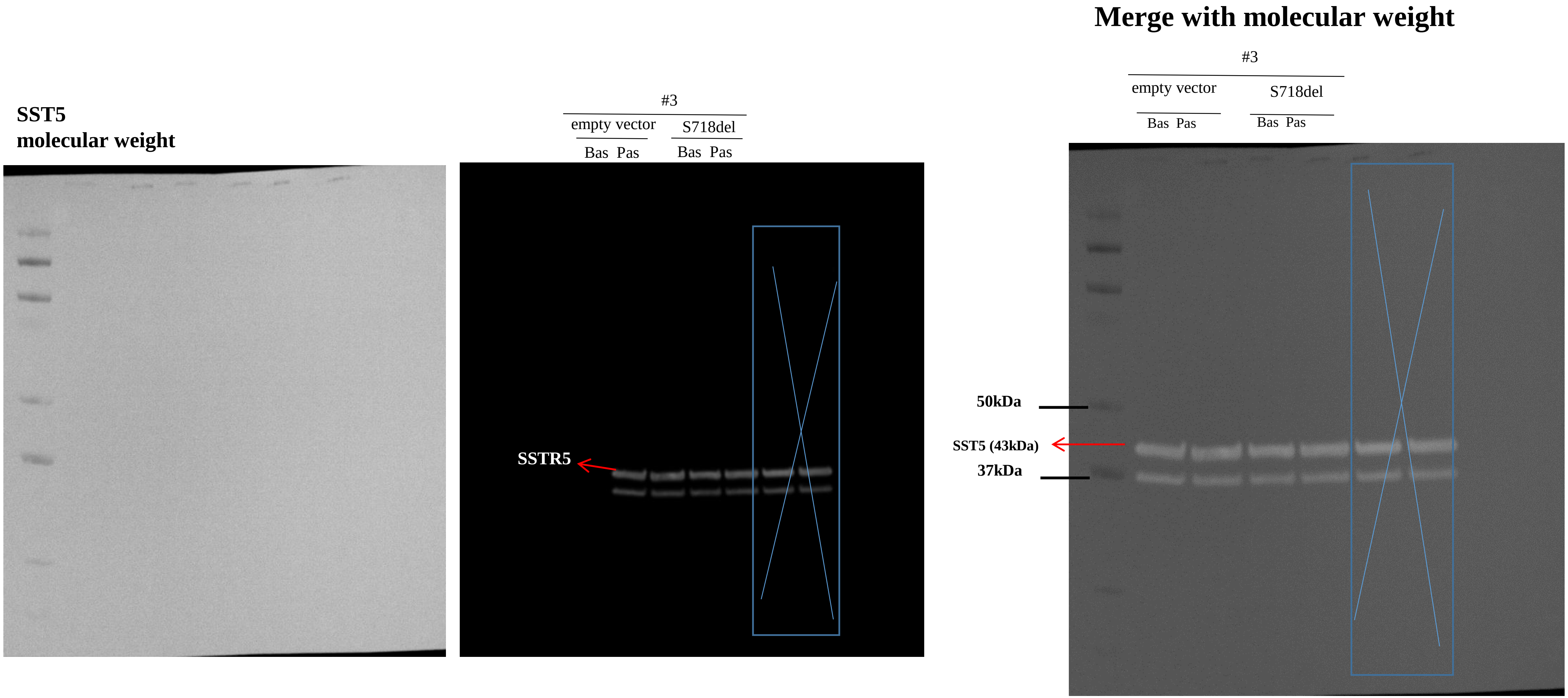

Fig. S1 i: Original western blot and merge with molecular weight. Immunoblot of SST5 in USP8 wild-type ACTH-secreting Pit-NET primary cultures transfected with USP8-718del mutant and treated with pasireotide. Membranes were incubated with SST5 antibody**.**

Fig. S2 a

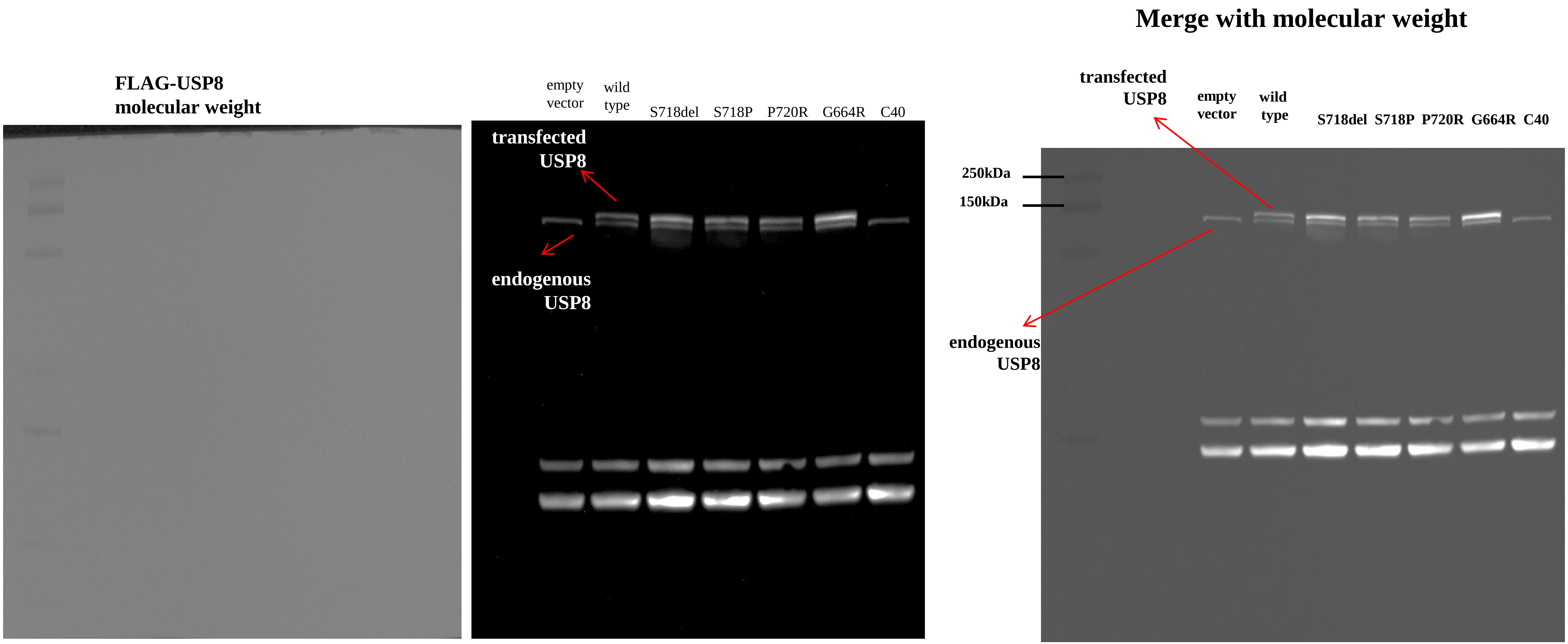

Fig. S2 a: Original western blot and merge with molecular weight. Immunoblot of USP8 in AtT-20 cells transfected with USP8 mutants. Membranes were incubated with USP8 antibody.

Fig. S2 b

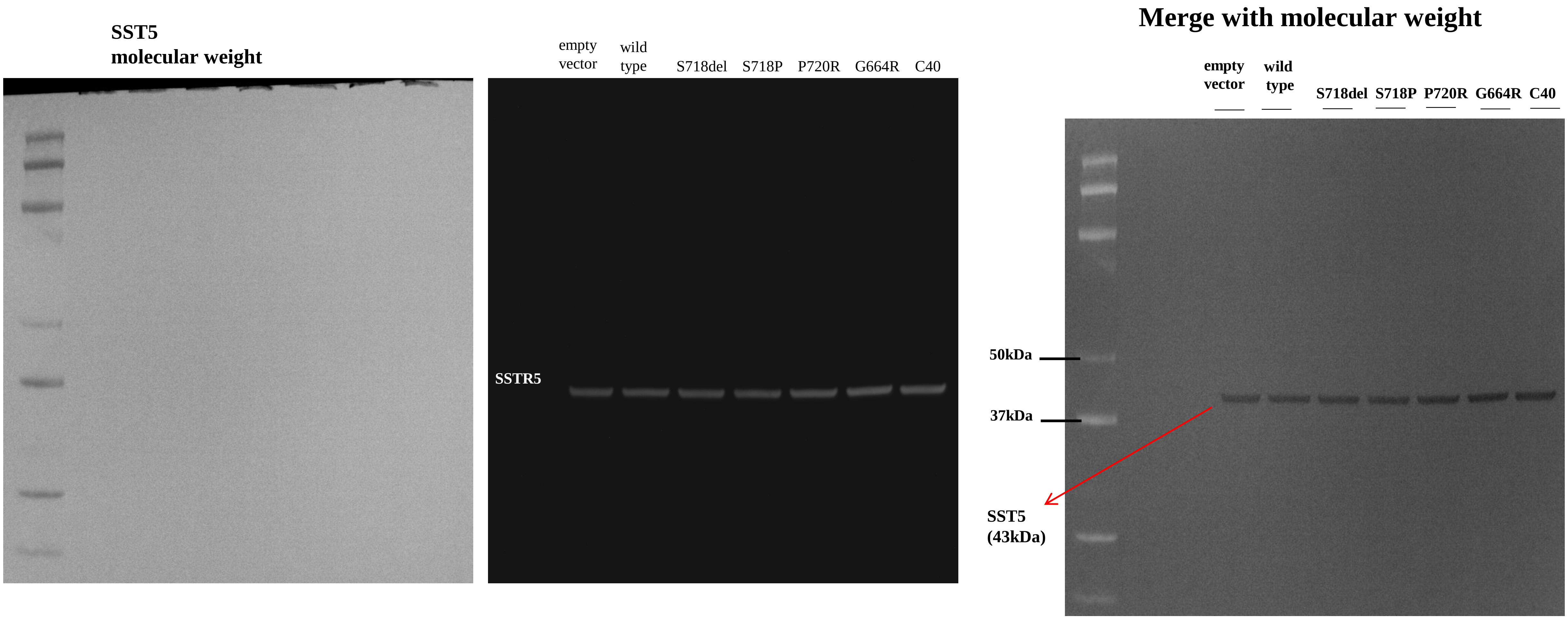

Fig. S2 b: Original western blot and merge with molecular weight. Immunoblot of SST5 in AtT-20 cells transfected with USP8 mutants. Membranes were incubated with SST5 antibody.

Fig. S2 c

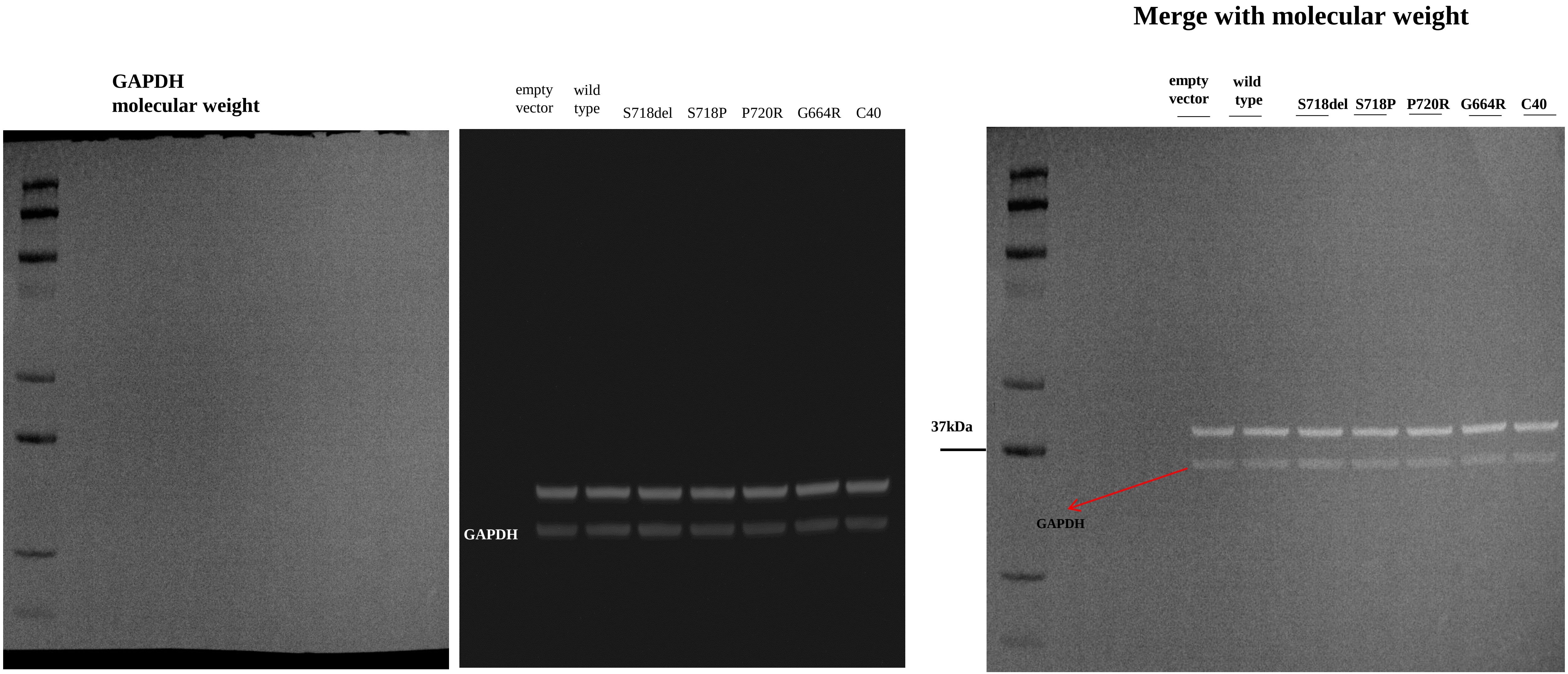

Fig. S2 c: Original western blot and merge with molecular weight. Immunoblot of GAPDH in AtT-20 cells transfected with USP8 mutants. Membranes were incubated with GAPDH antibody.

Fig. S2 d

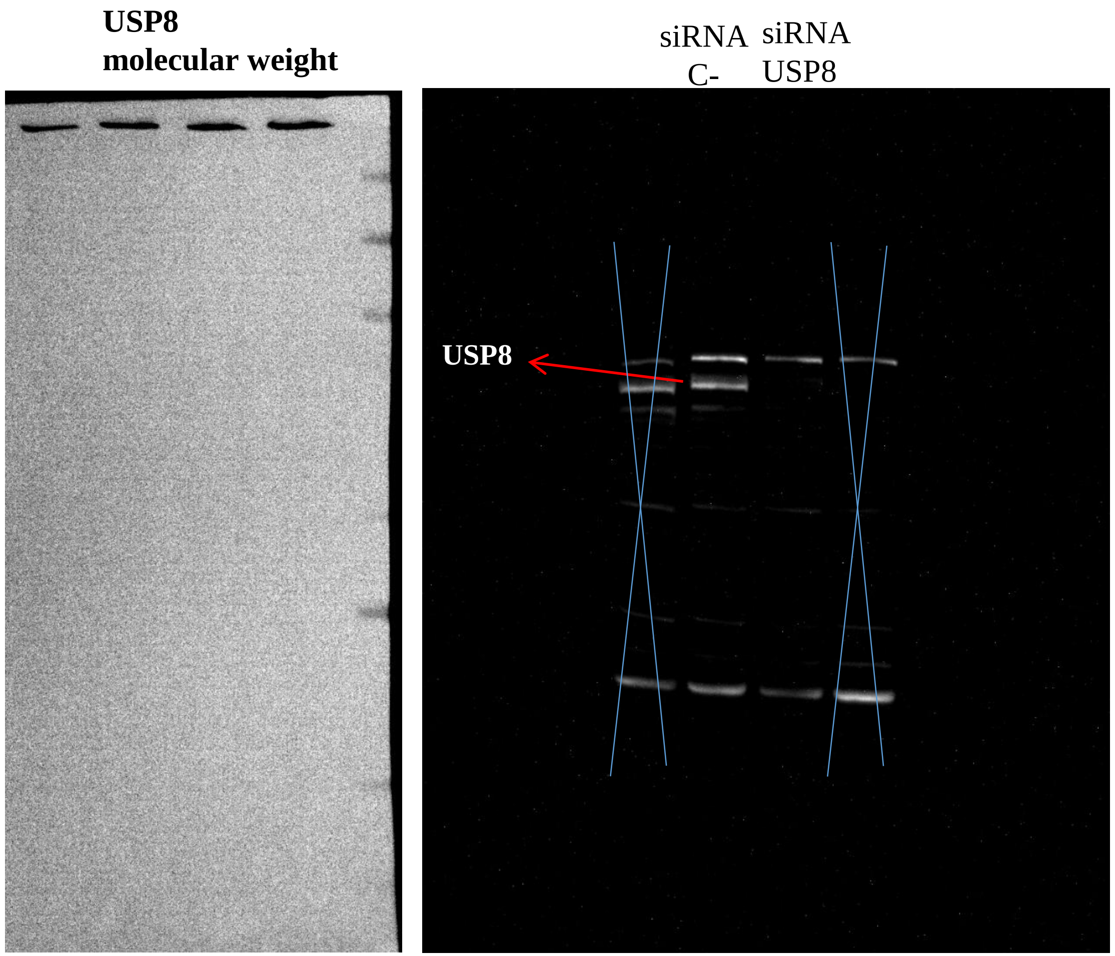

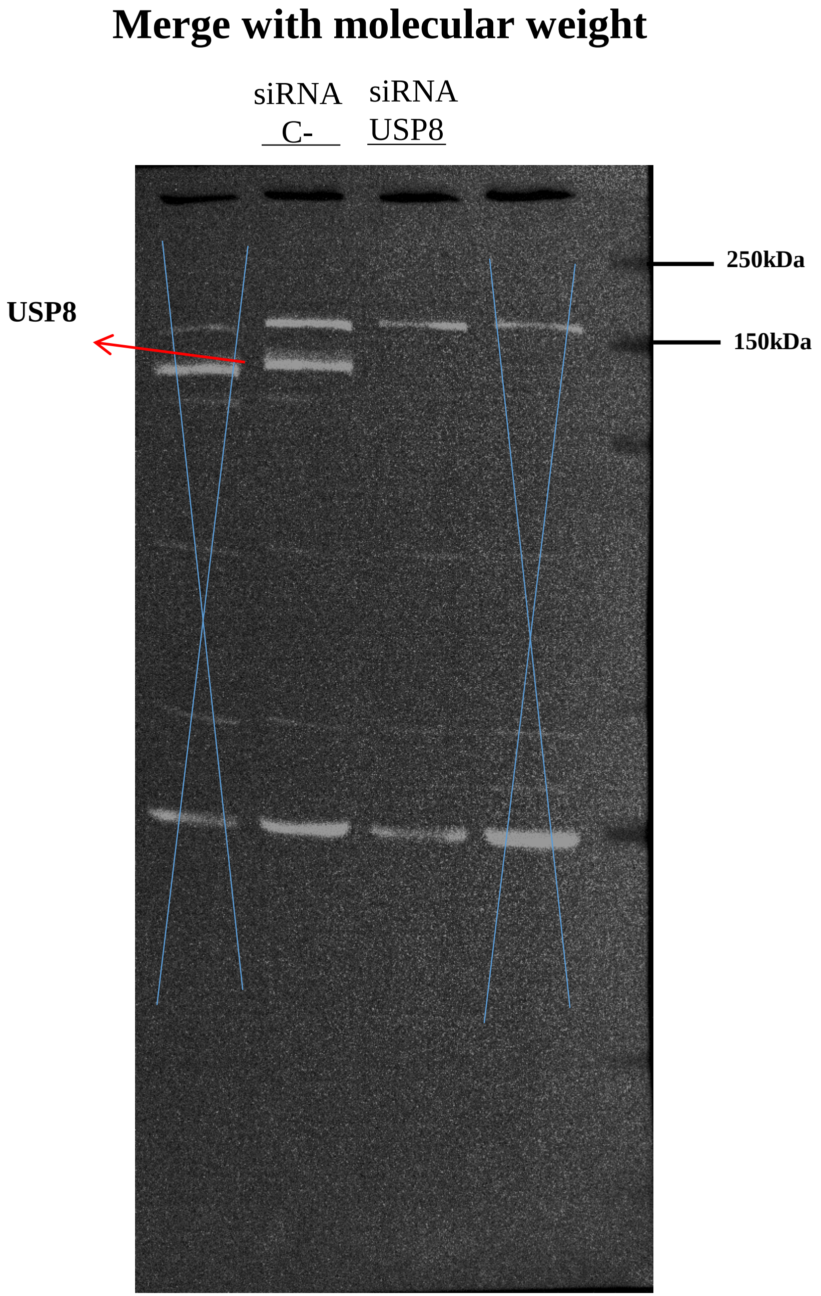

Fig. S2 d Original western blot and merge with molecular weight. Immunoblot of USP8 in AtT-20 cells silenced with USP8 mutant. Membranes were incubated with USP8 antibody.

Fig. S2 e

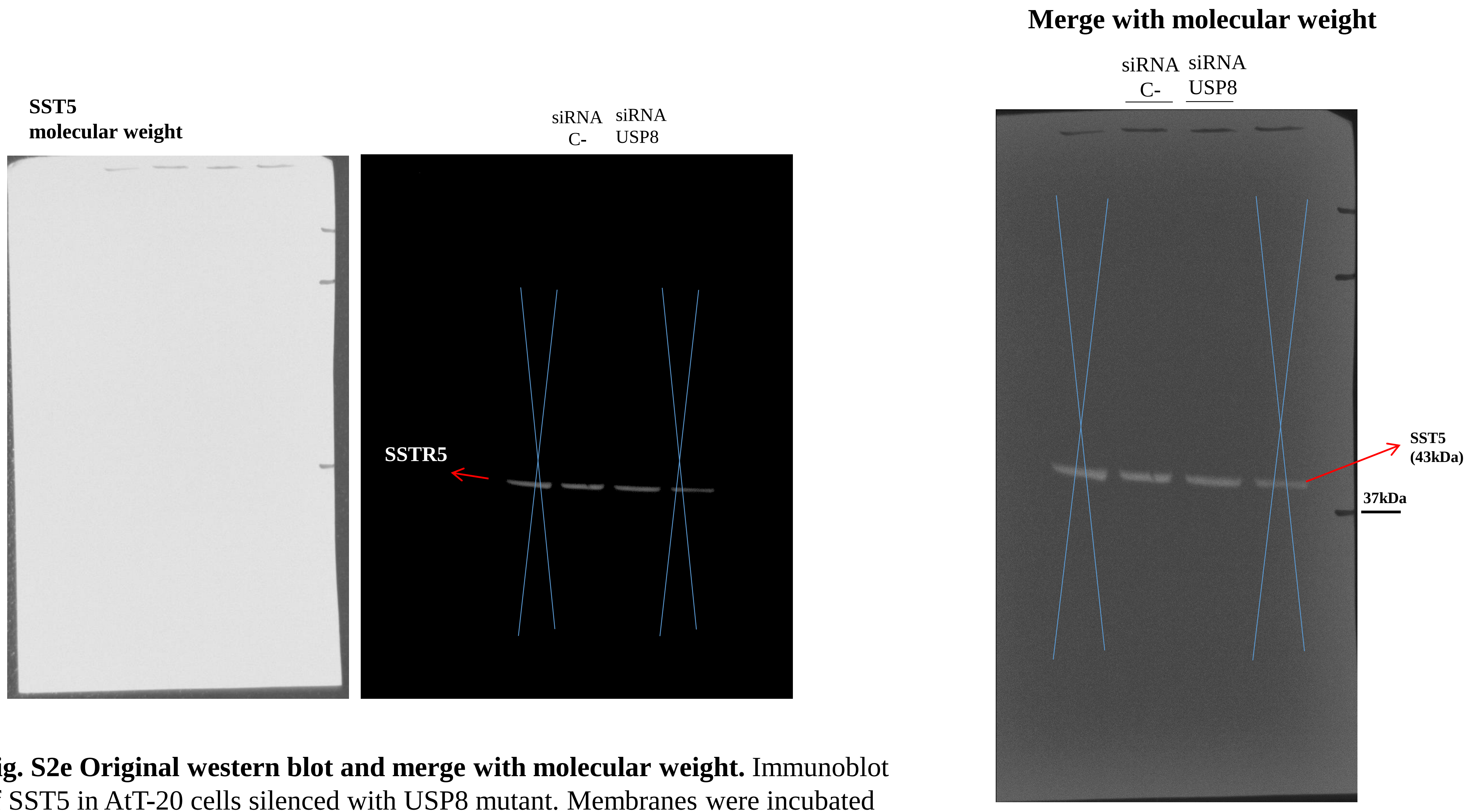

Fig. S2e Original western blot and merge with molecular weight. Immunoblot of SST5 in AtT-20 cells silenced with USP8 mutant. Membranes were incubated with SST5 antibody.

Fig. S2 f

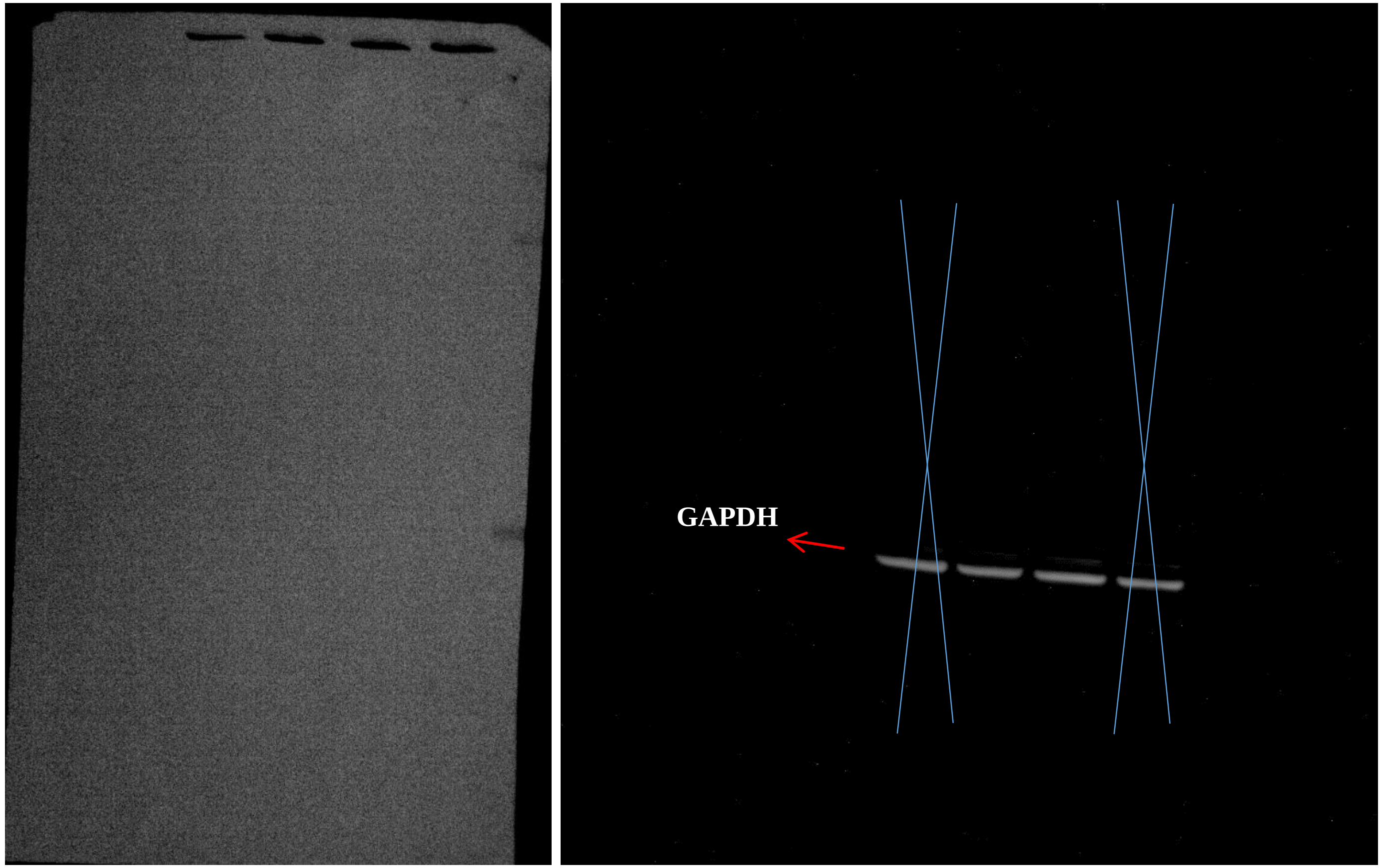

Merge with molecular weight

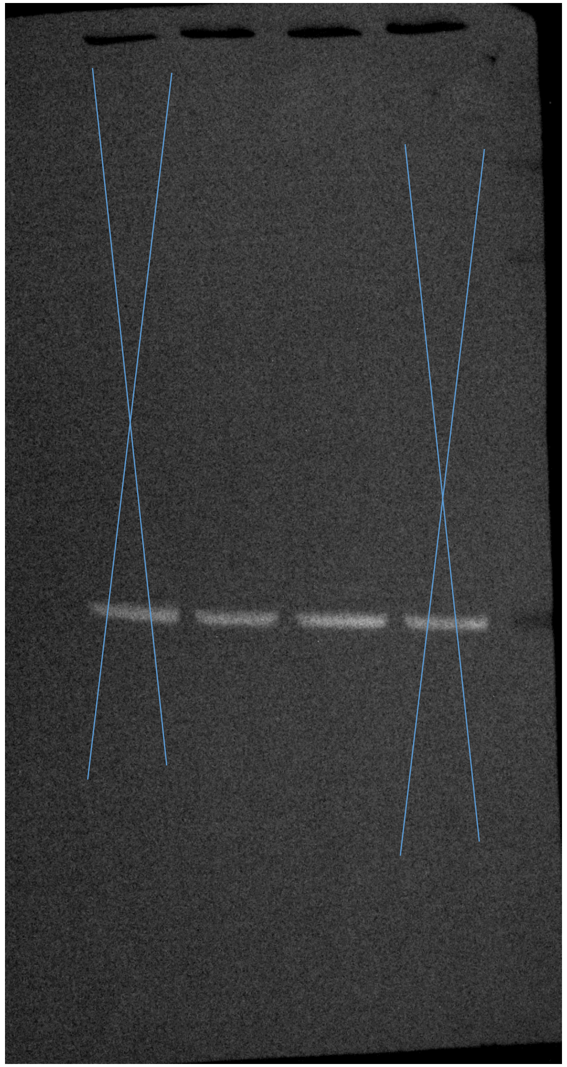

Fig. S2 f Original western blot and merge with molecular weight. Immunoblot of GAPDH in AtT-20 cells silenced with USP8 mutant. Membranes were incubated with GAPDH antibody.

Fig. S3 a

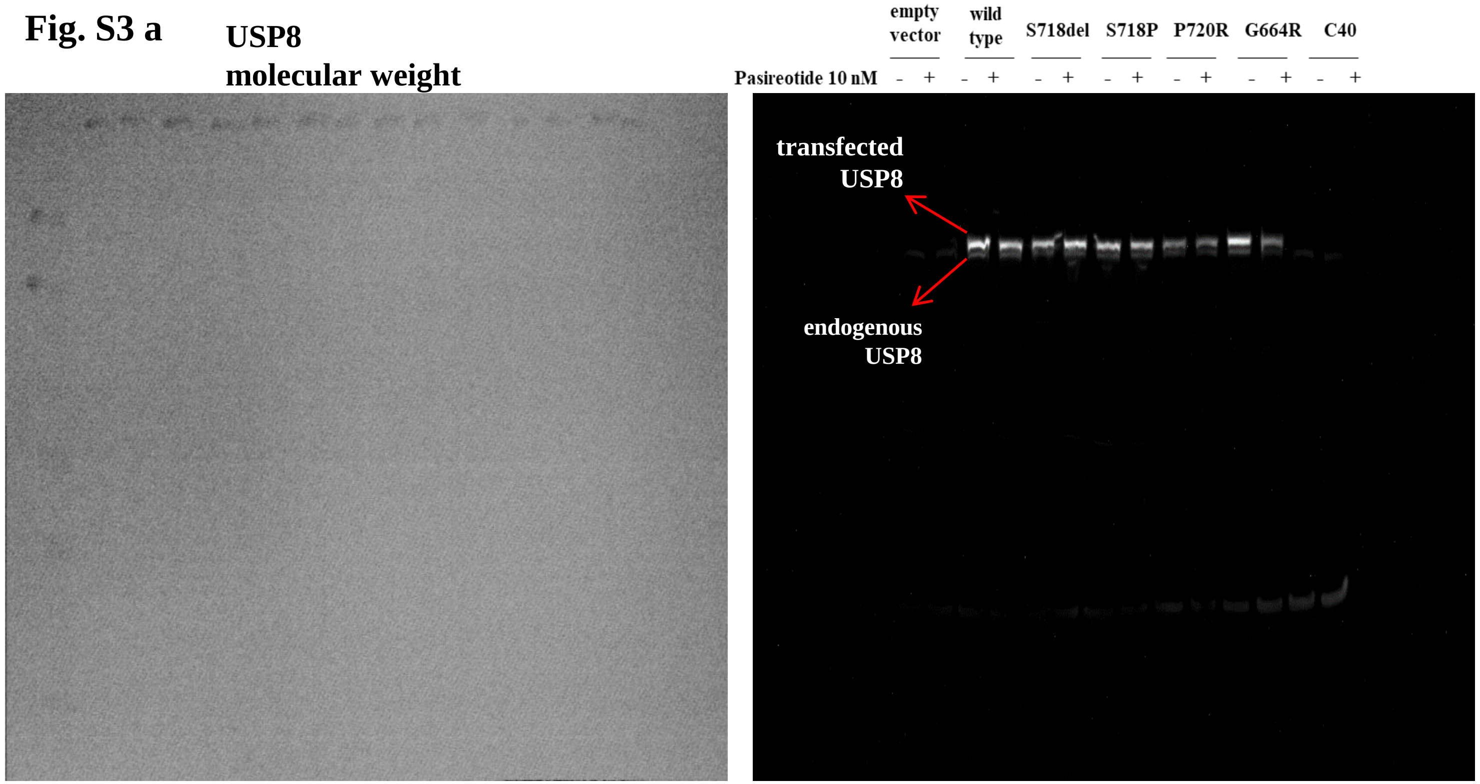

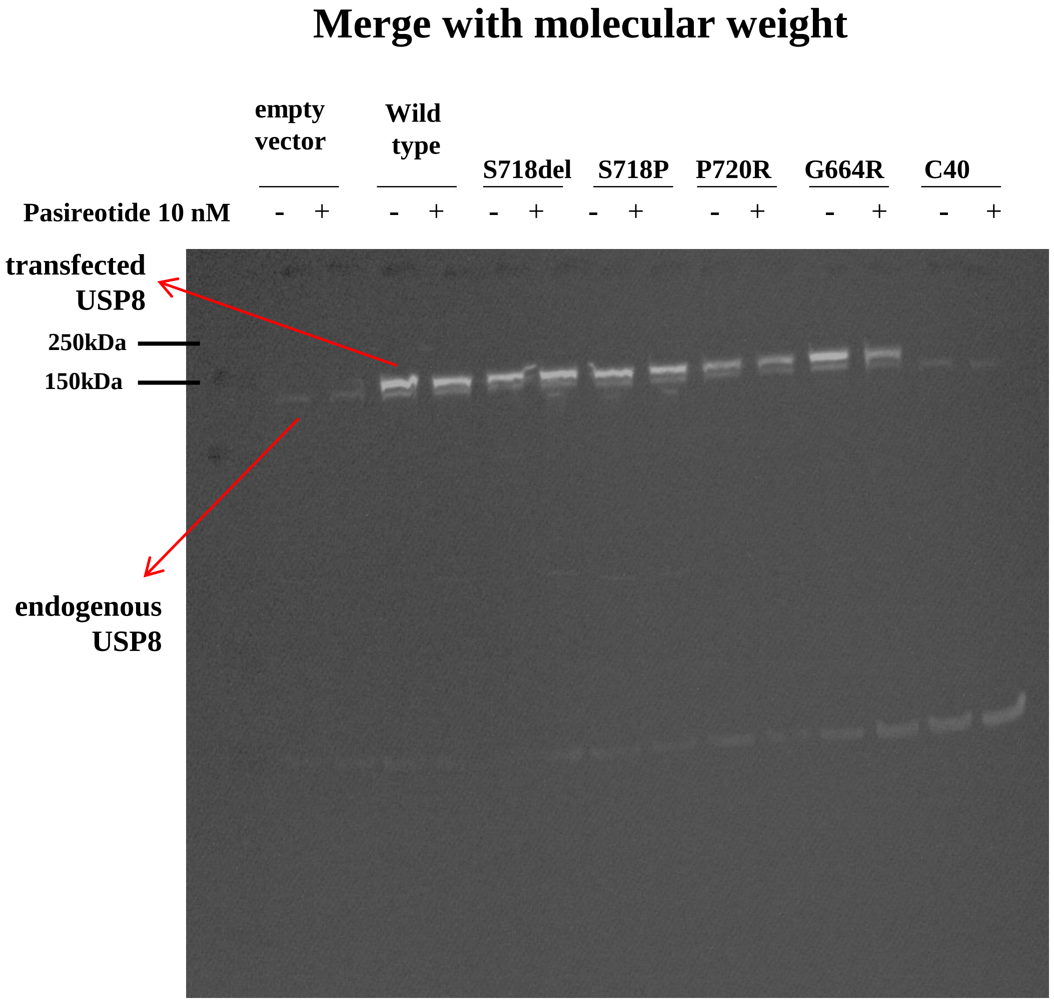

Fig. S3 a Original western blot and merge with molecular weight. Immunoblot of USP8 in AtT-20 cells transfected with USP8 mutants and treated with pasireotide. Membranes were incubated with USP8 antibody.

Fig. S3 b

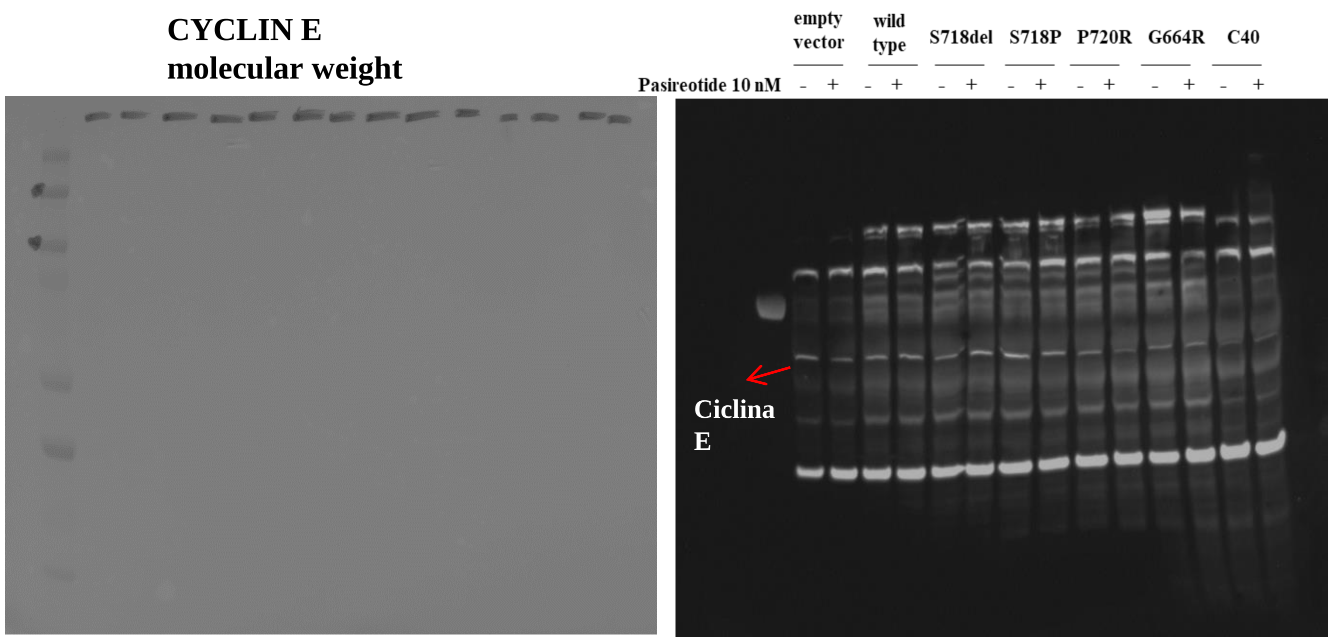

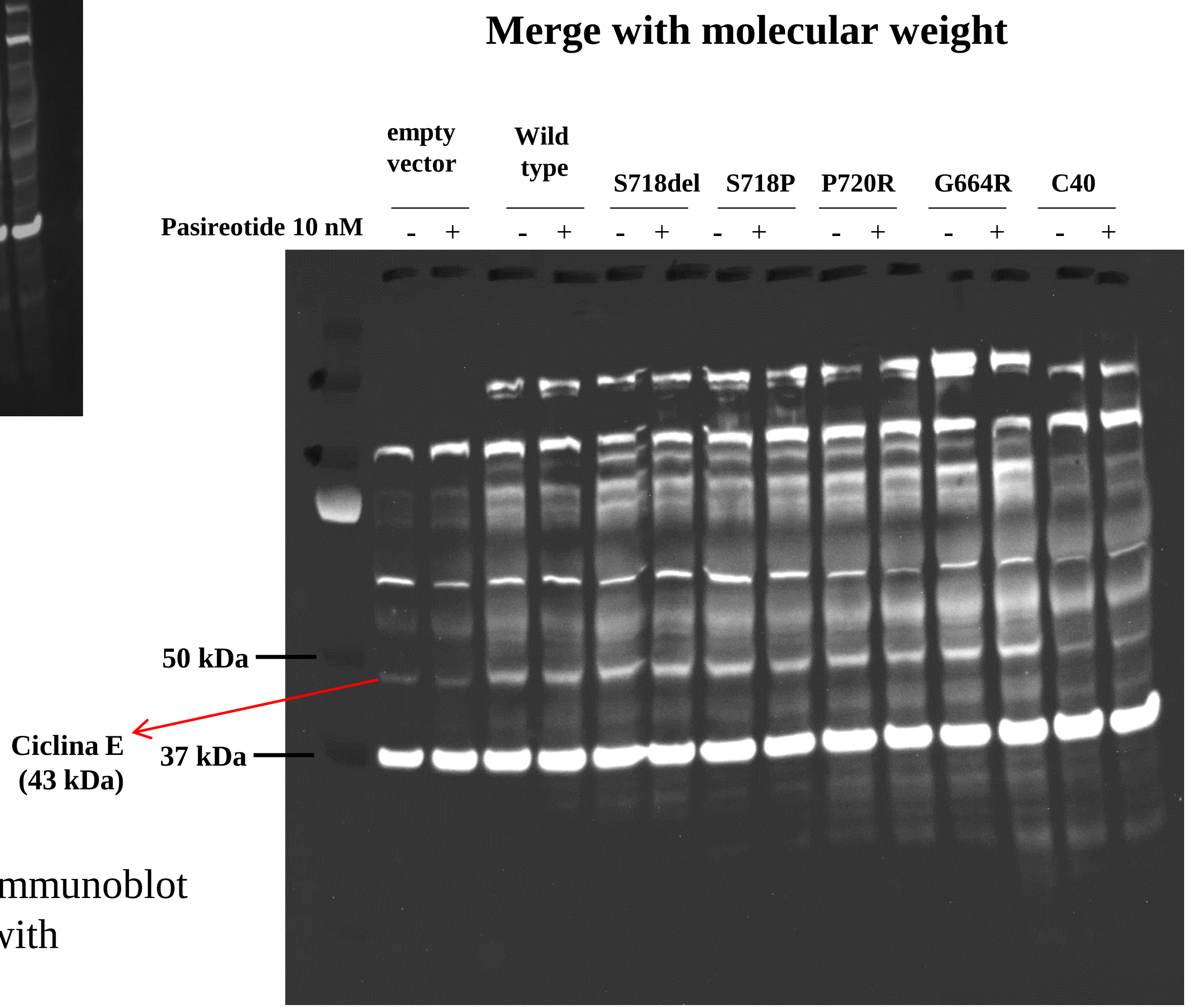

Fig. S3 b Original western blot and merge with molecular weight. Immunoblot of cyclin E in AtT-20 cells transfected with USP8 mutants and treated with pasireotide. Membranes were incubated with cyclin E antibody.

Fig. S3 c

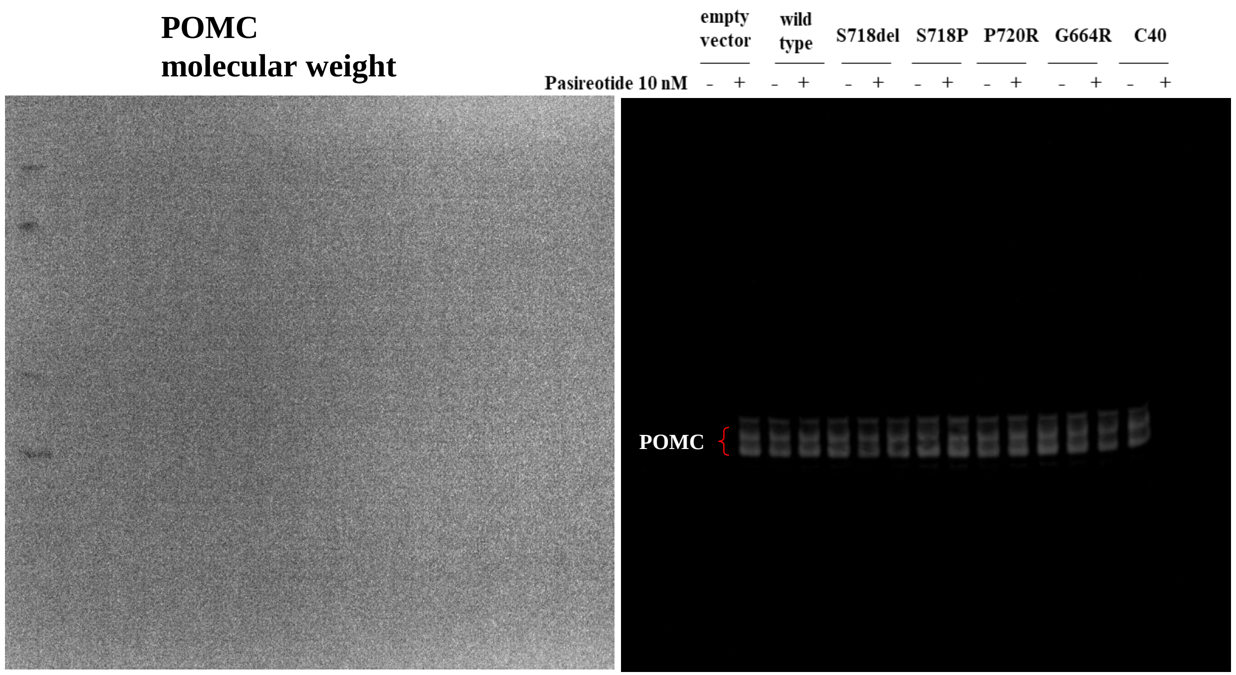

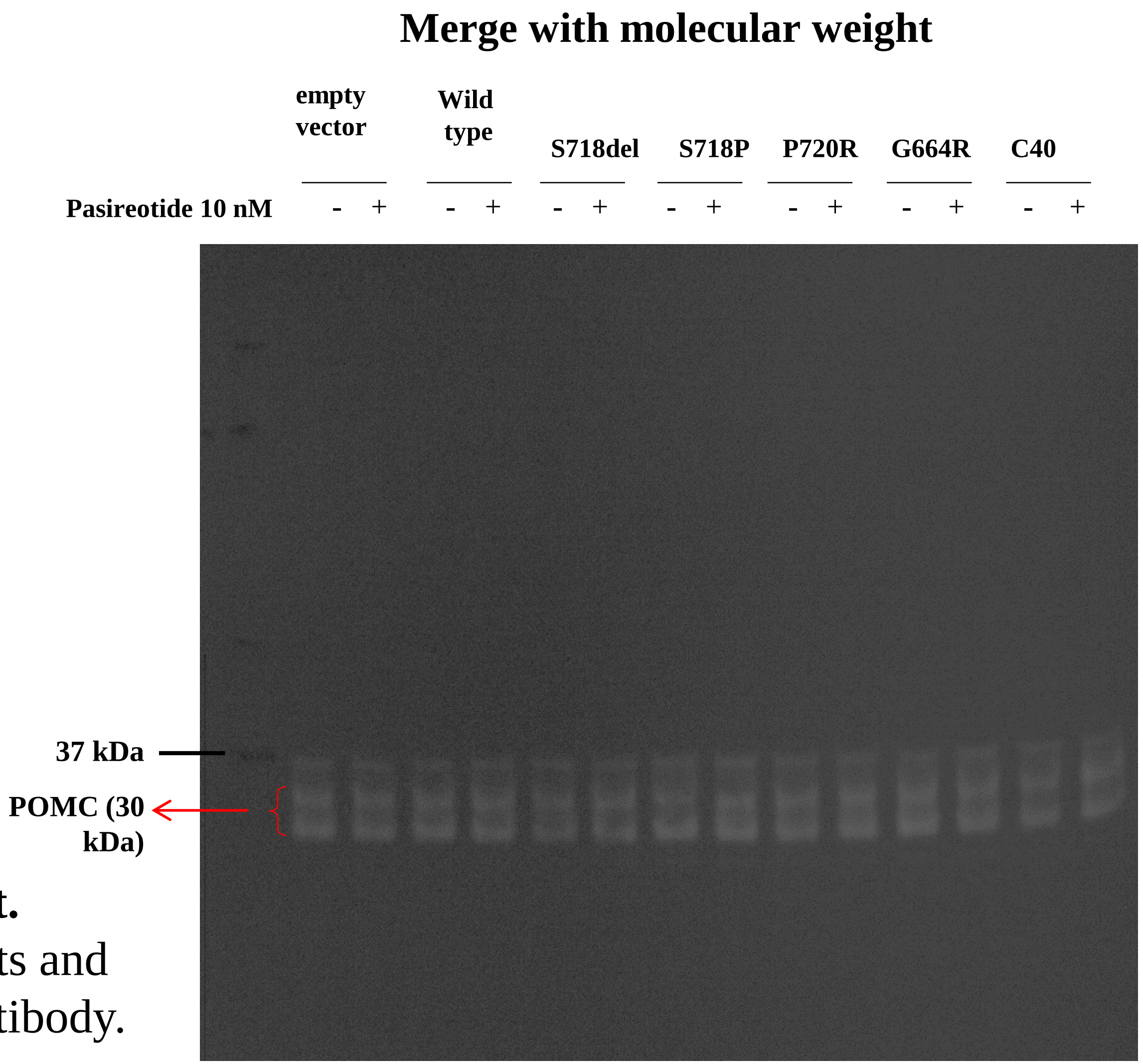

Fig. S3 c Original western blot and merge with molecular weight. Immunoblot of POMC in AtT-20 cells transfected with USP8 mutants and treated with pasireotide. Membranes were incubated with POMC antibody.

Fig. S3 d

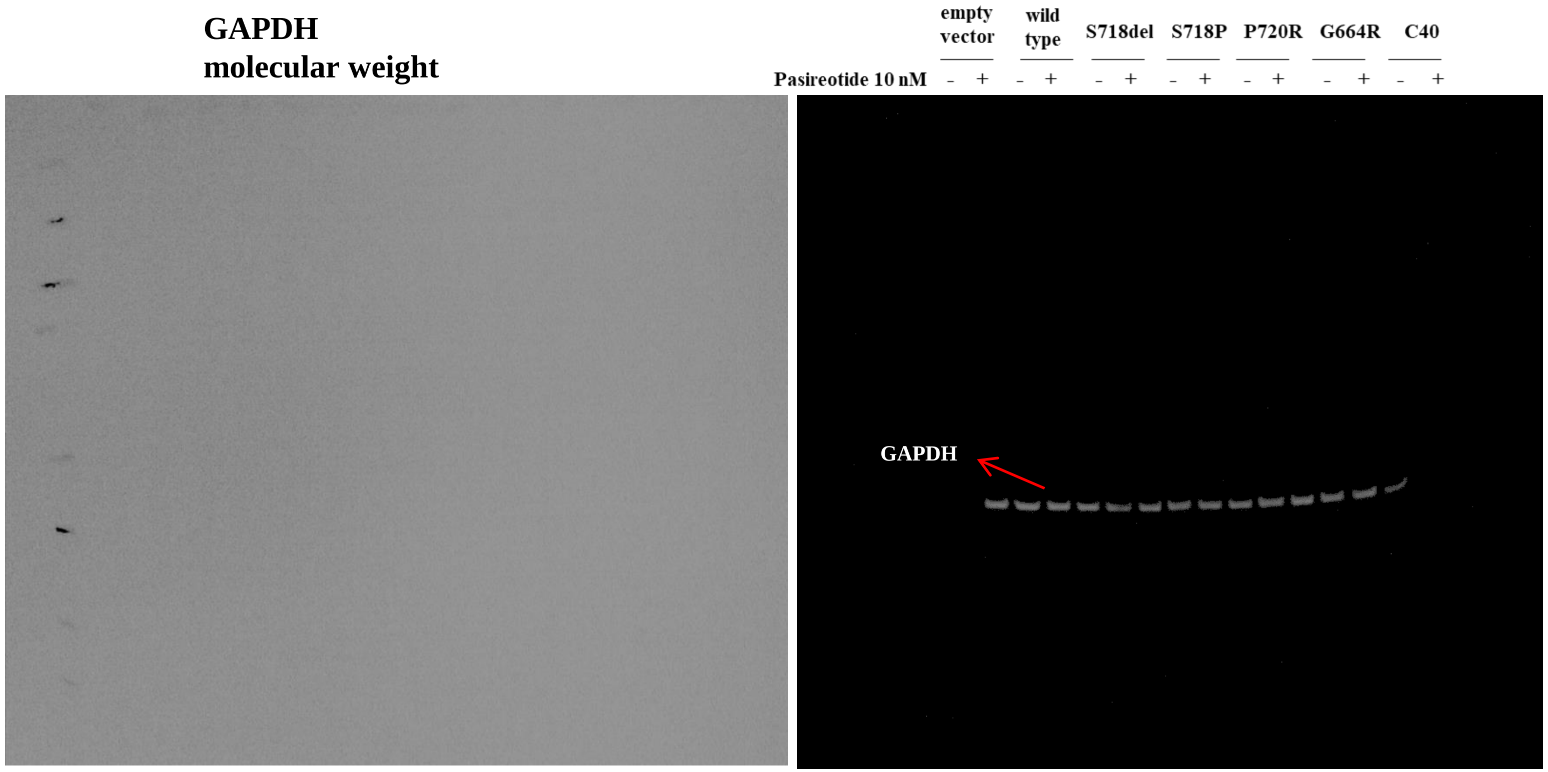

Merge with molecular weight

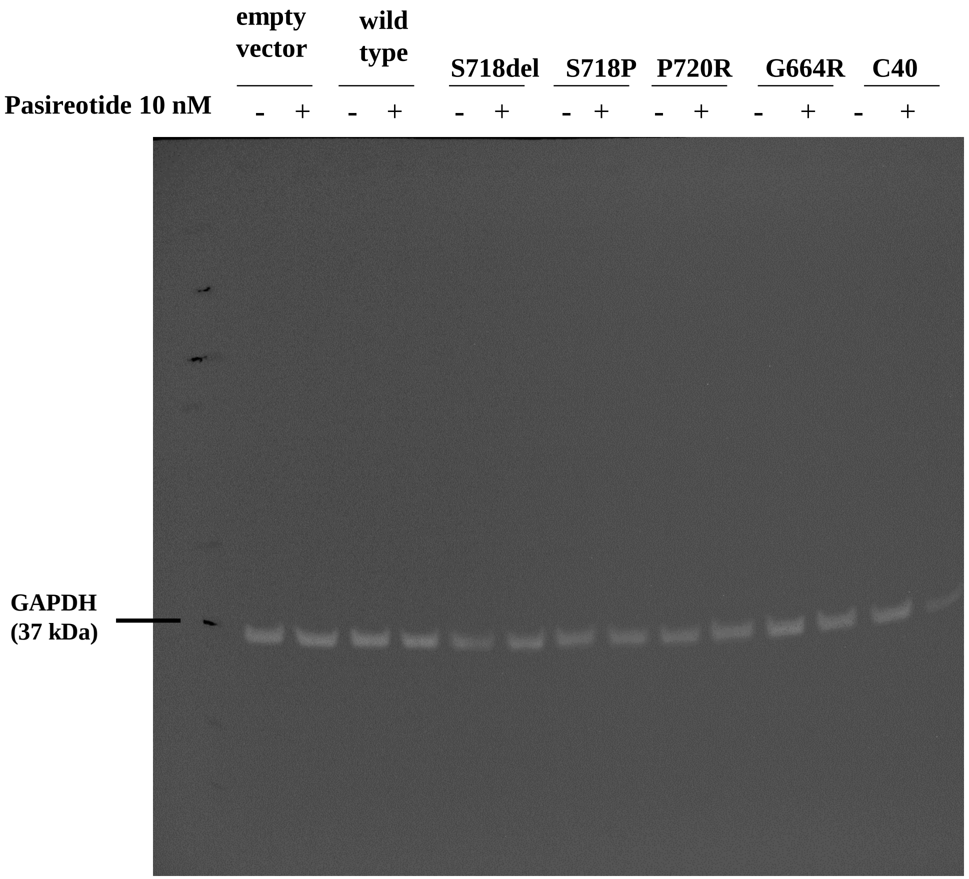

Fig. S3 d Original western blot and merge with molecular weight. Immunoblot of GAPDH in AtT-20 cells transfected with USP8 mutants and treated with pasireotide. Membranes were incubated with GAPDH antibody.

Fig. S4 a

CD3
molecular weight

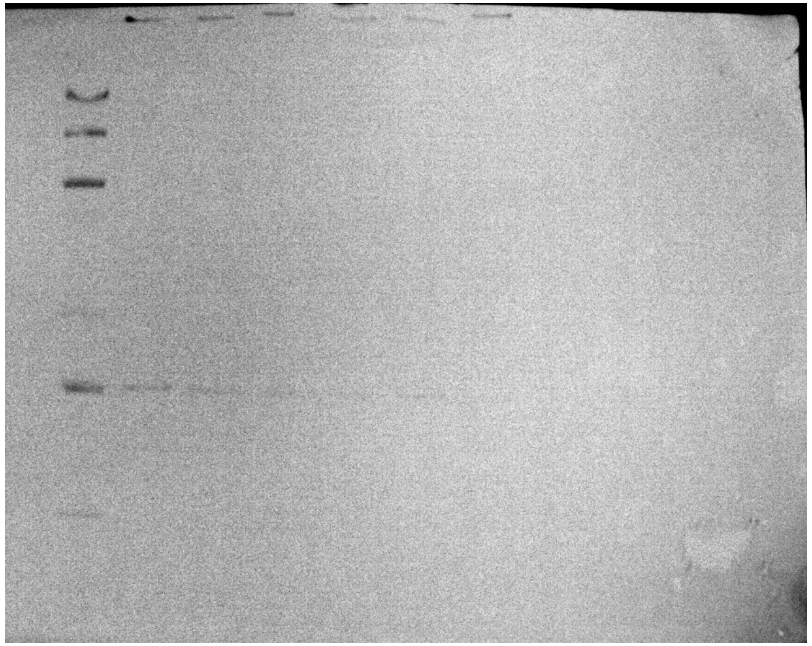

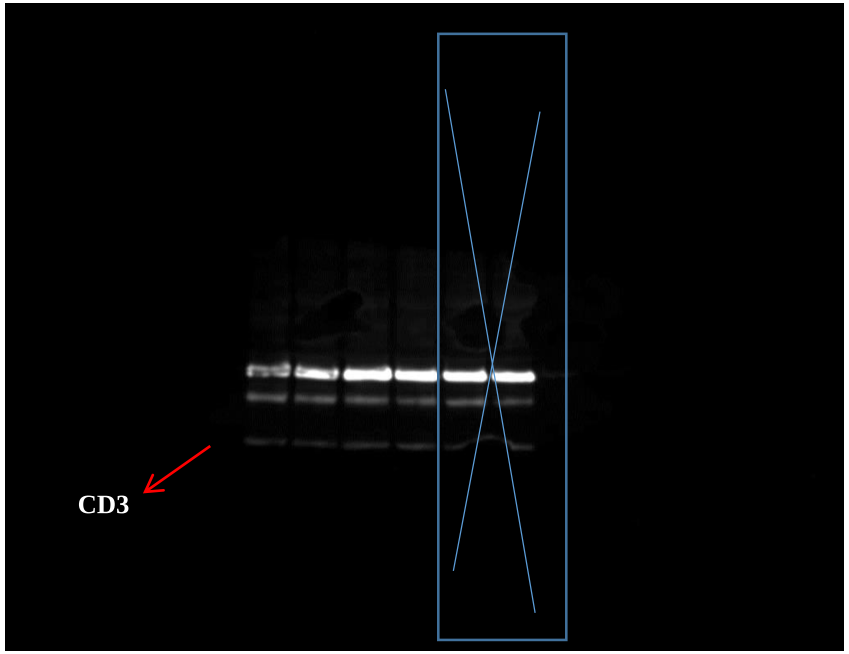

Merged with molecular weight

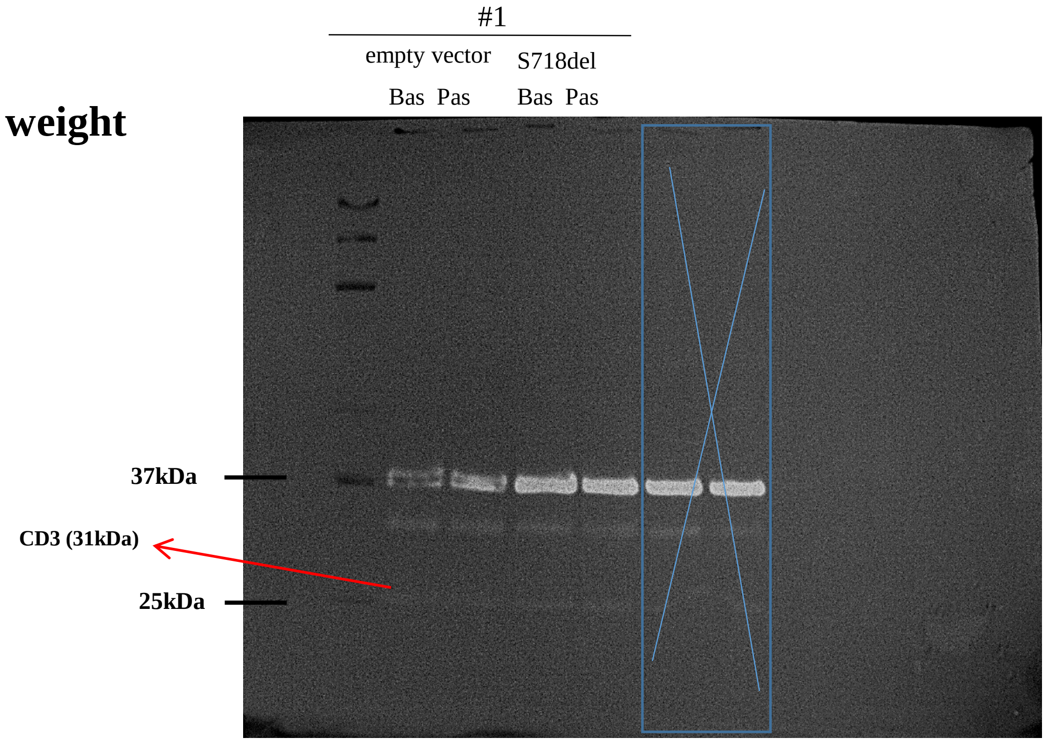

Fig. S4 a: Original western blot and merge with molecular weight. Immunoblot of CD3 and GAPDH in USP8 wild-type ACTH-secreting Pit-NET primary cultures transfected with USP8-718del mutant and treated with pasiretoide. Membranes were incubated with CD3 and GAPDH antibodies.

Fig. S4 b

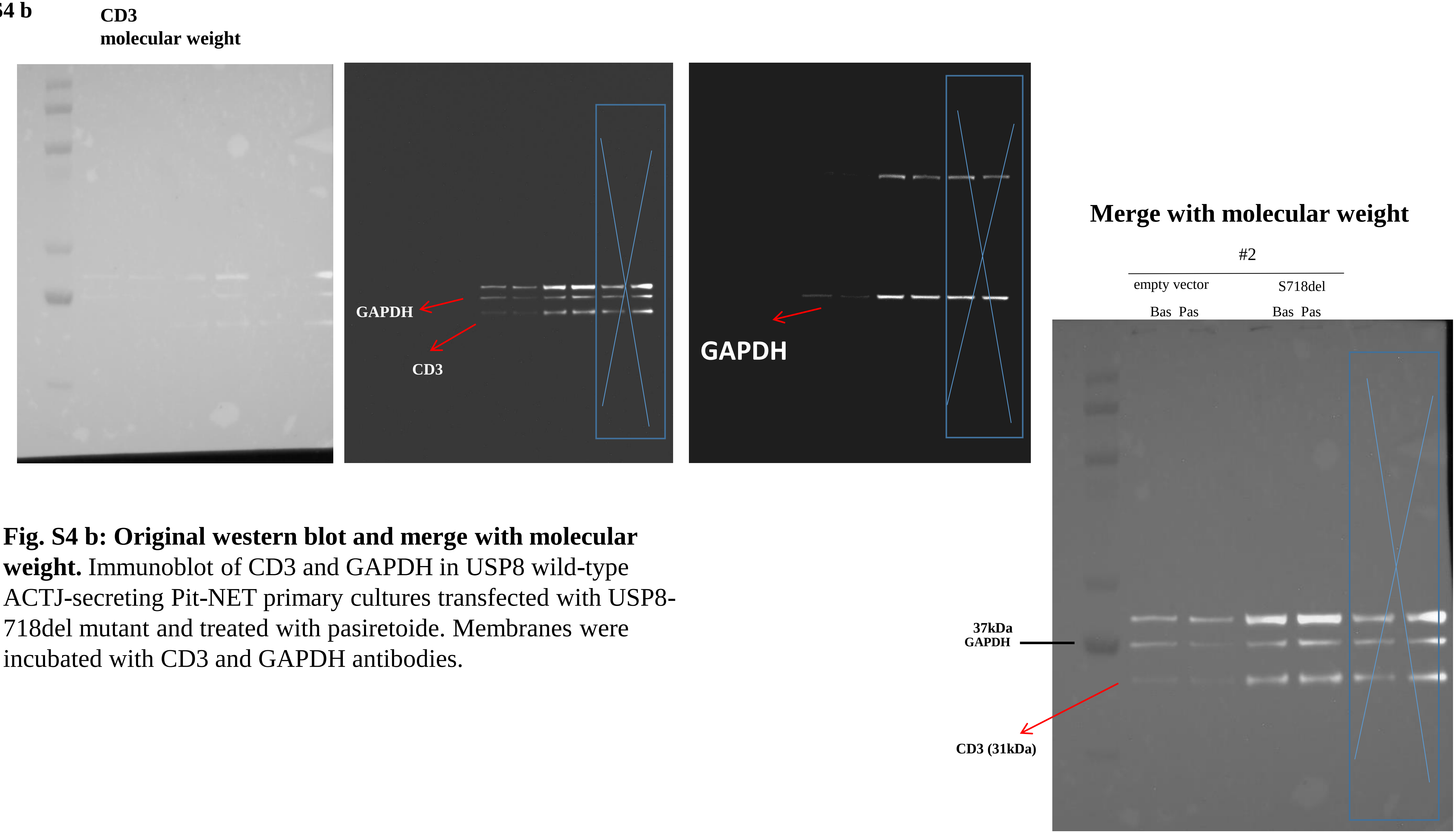

Fig. S4 b: Original western blot and merge with molecular weight. Immunoblot of CD3 and GAPDH in USP8 wild-type ACTJ-secreting Pit-NET primary cultures transfected with USP8-718del mutant and treated with pasiretoide. Membranes were incubated with CD3 and GAPDH antibodies.

Fig. S4 c

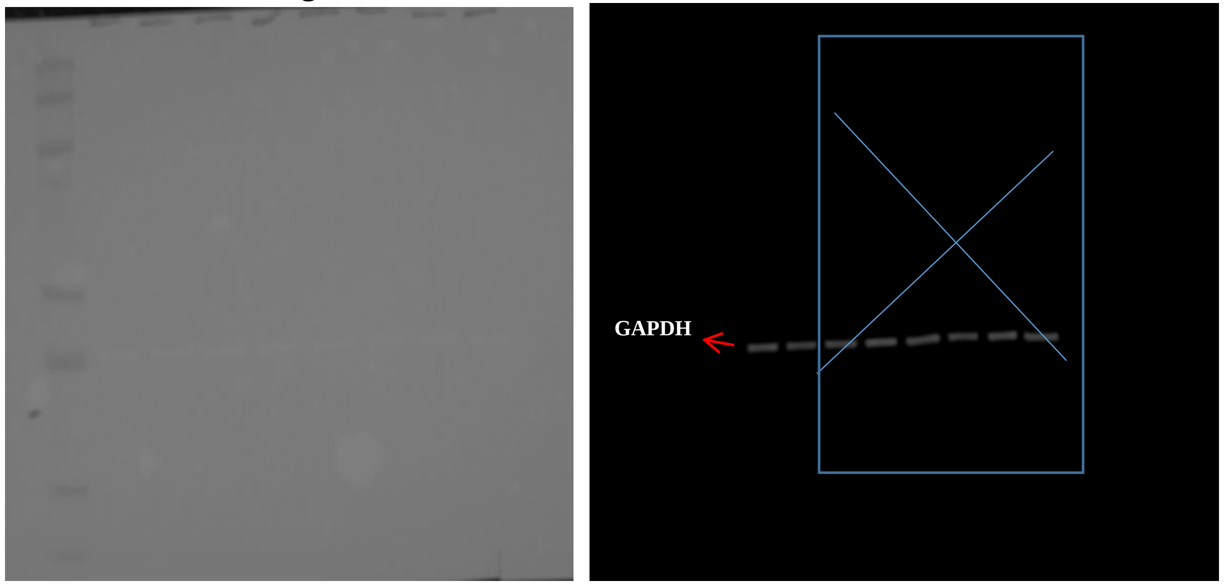

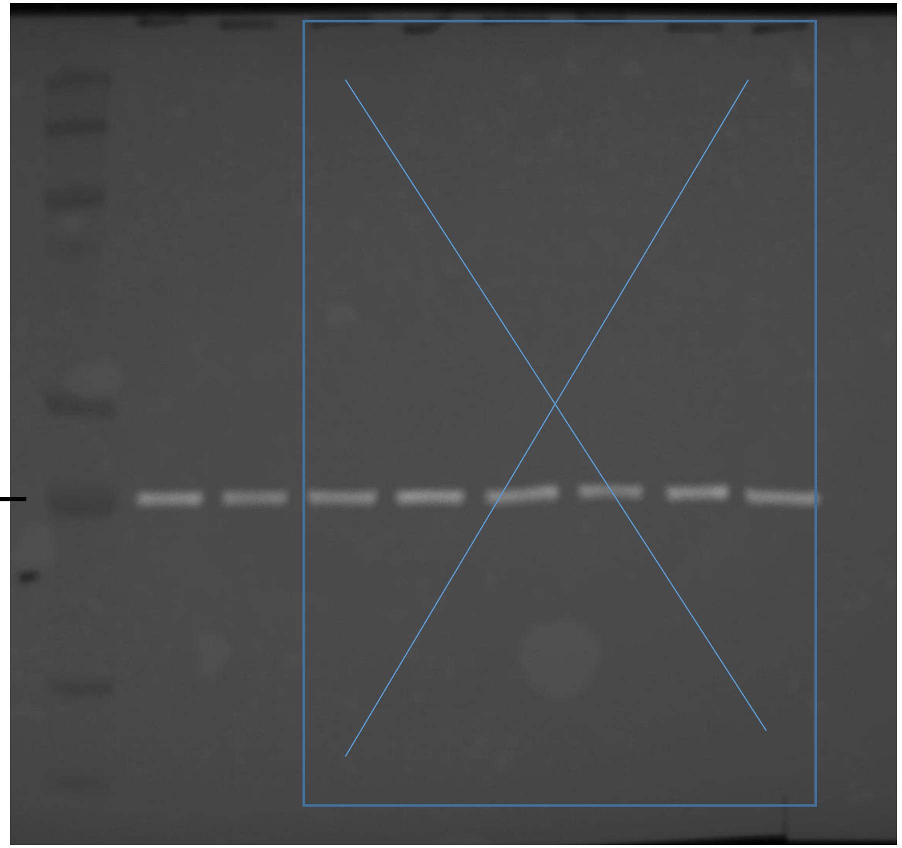

Fig. S4 c Original western blot and merge with molecular weight. Immunoblot of GAPDH in P720 USP8-mutated ACTH-secreting Pit-NET primary culture treated with pasireotide. Membranes were incubated with GAPDH antibody.

Fig. S4 d

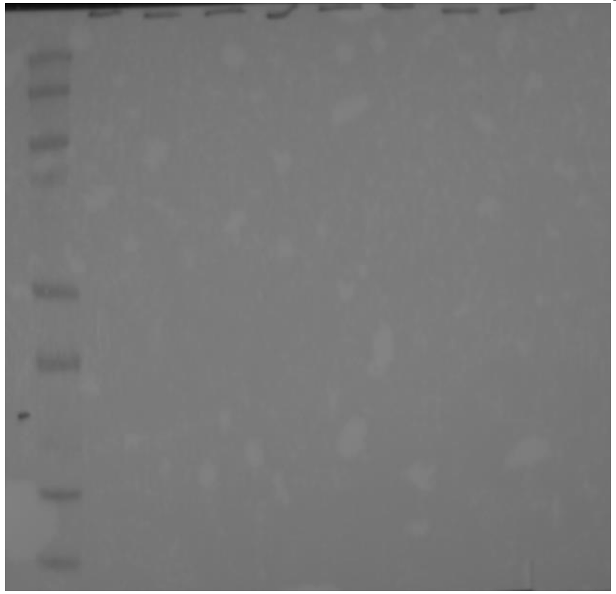

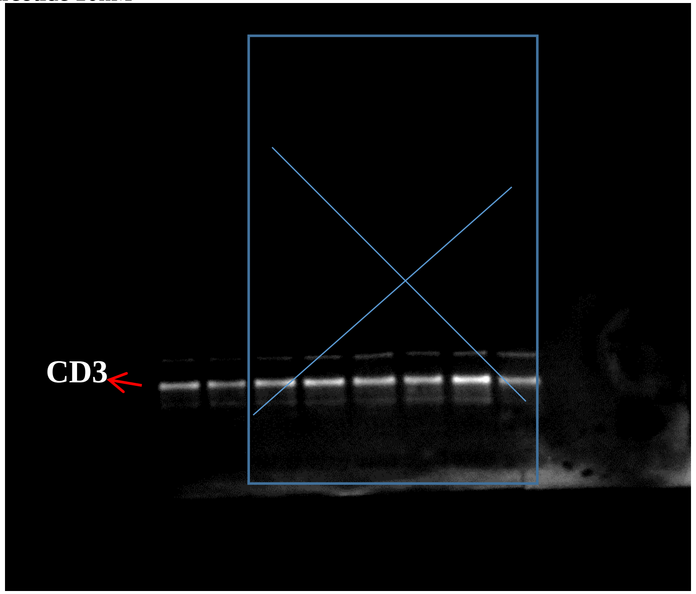

Merge with molecular weight

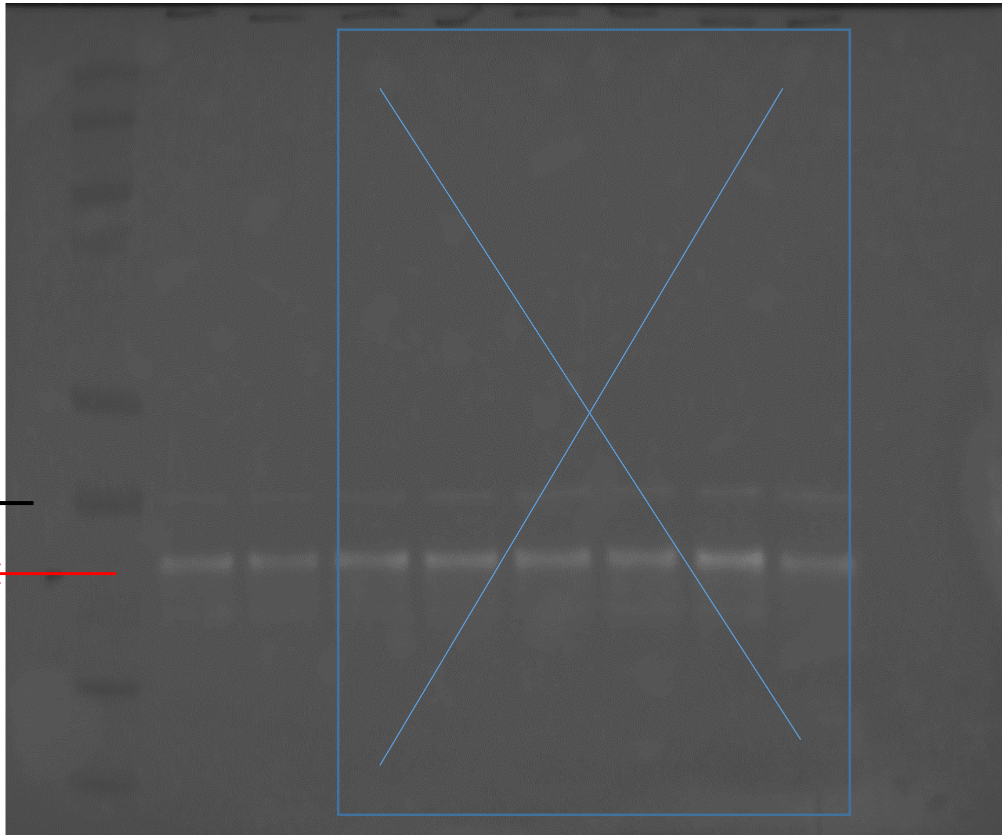

Fig. S4 d Original western blot and merge with molecular weight. Immunoblot of CD3 in P720 USP8-mutated ACTH-secreting Pit-NET primary culture treated with pasireotide. Membranes were incubated with CD3 antibody.

Fig. S5 a

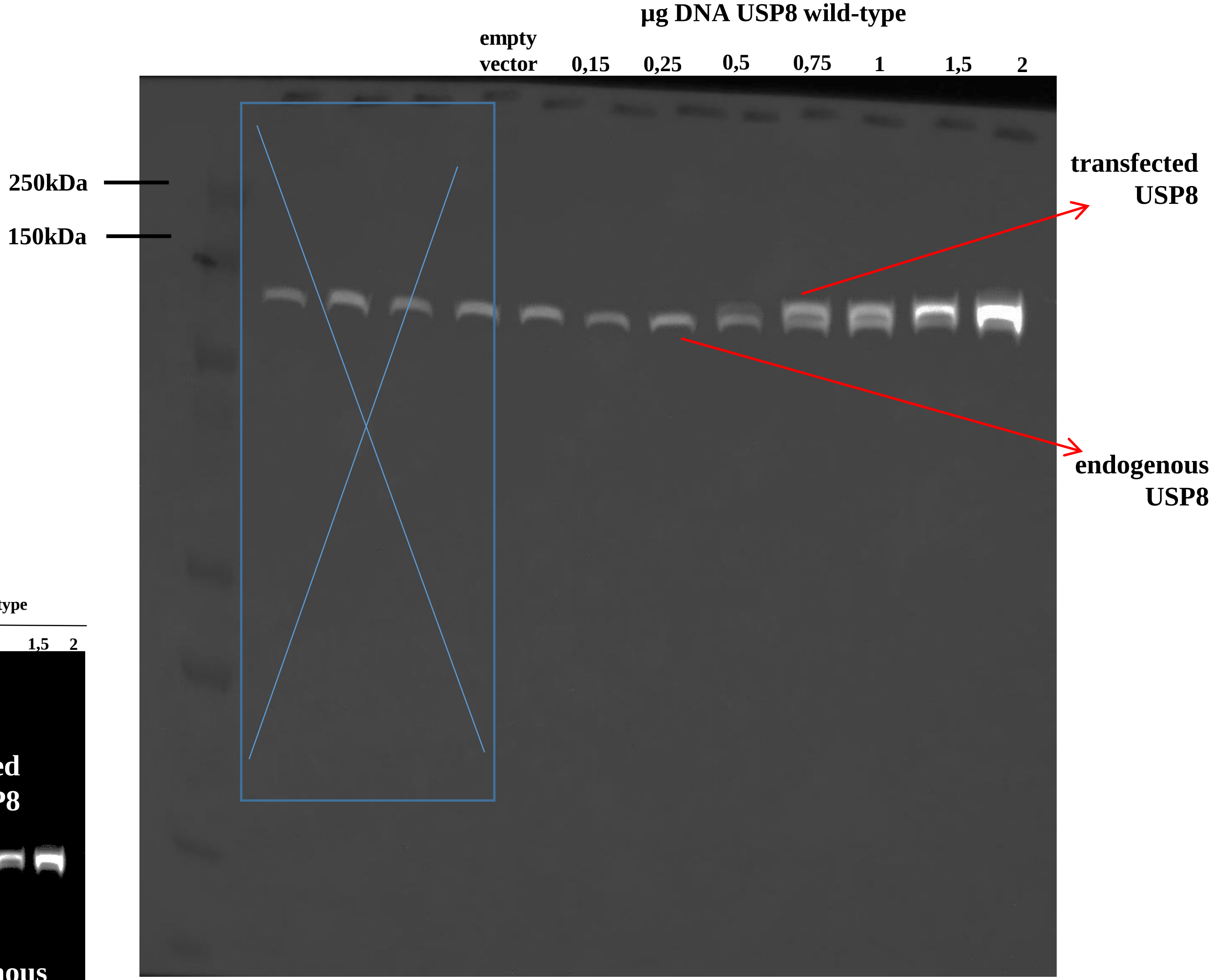

Merge with molecular weight

Fig. S5 a Original western blot and merge with molecular weight. Immunoblot of USP8 in AtT-20 cells transfected with different concentrations of USP8 wild type. Membranes were incubated with USP8 antibody.

USP8 molecular weight

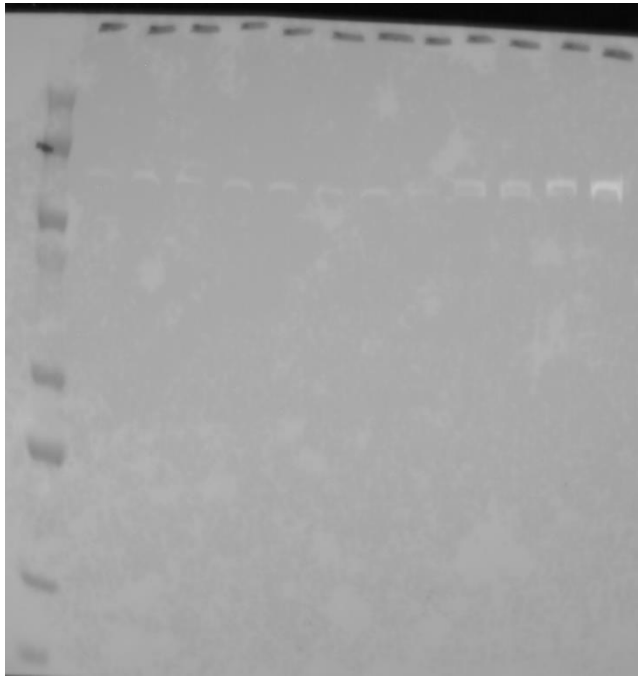

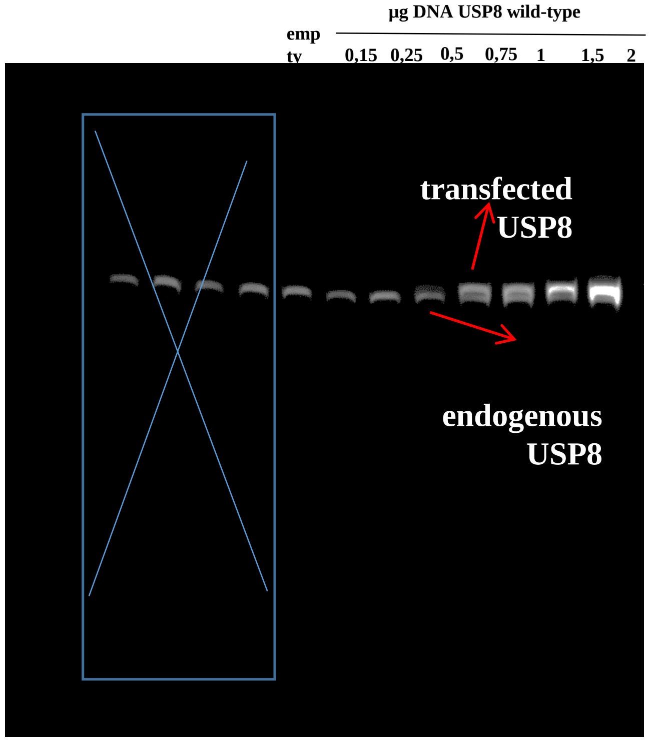

Fig. S5 b

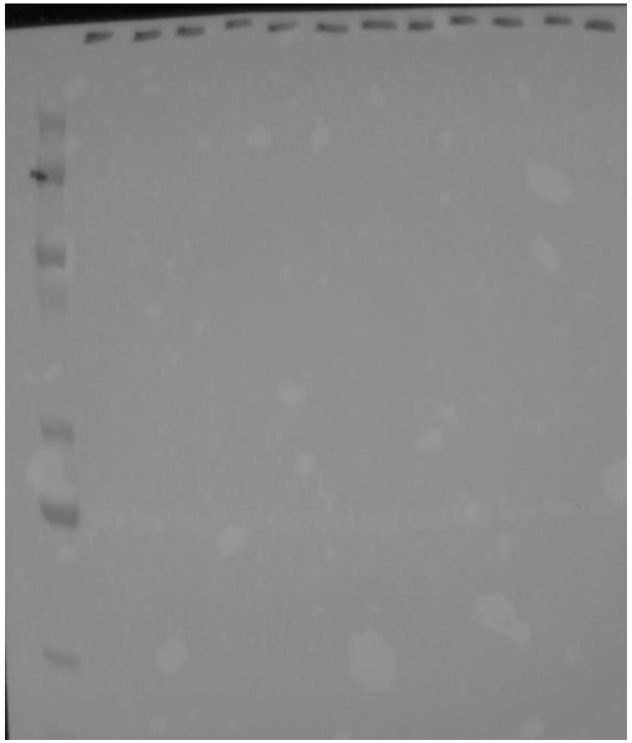

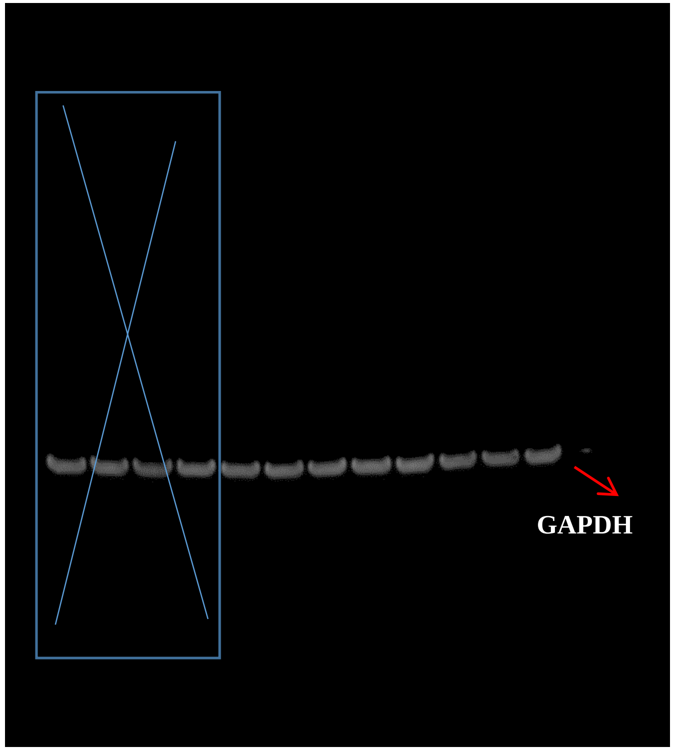

Merge with molecular weight

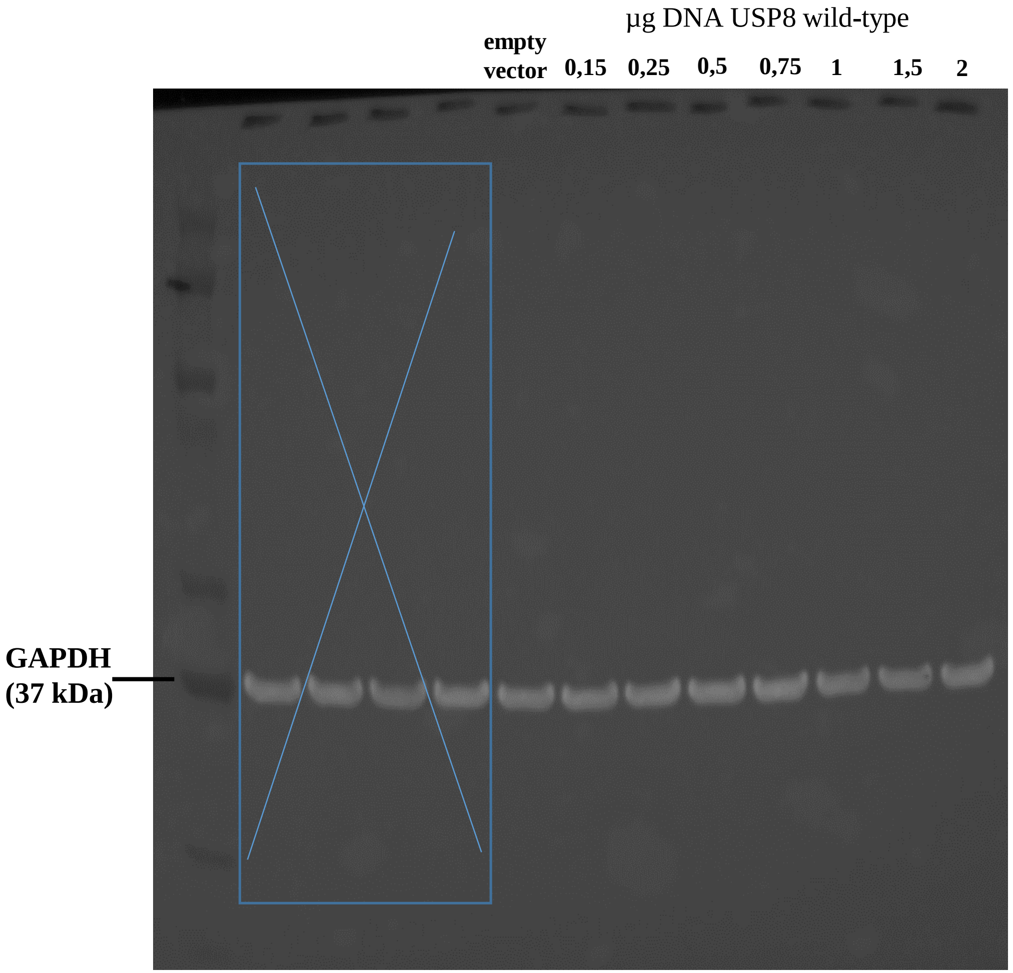

Fig. S5 b Original western blot and merge with molecular weight. Immunoblot of GAPDH in AtT-20 cells transfected with different concentrations of USP8 wild type. Membranes were incubated with GAPDH antibody.

Fig. S6 a

Fig. S6 a Original western blot and merge with molecular weight. Immunoblot of USP8 in AtT-20 cells transfected with USP8 wild type and treated with pasireotide. Membranes were incubated with USP8 antibody.

Merge with molecular weight

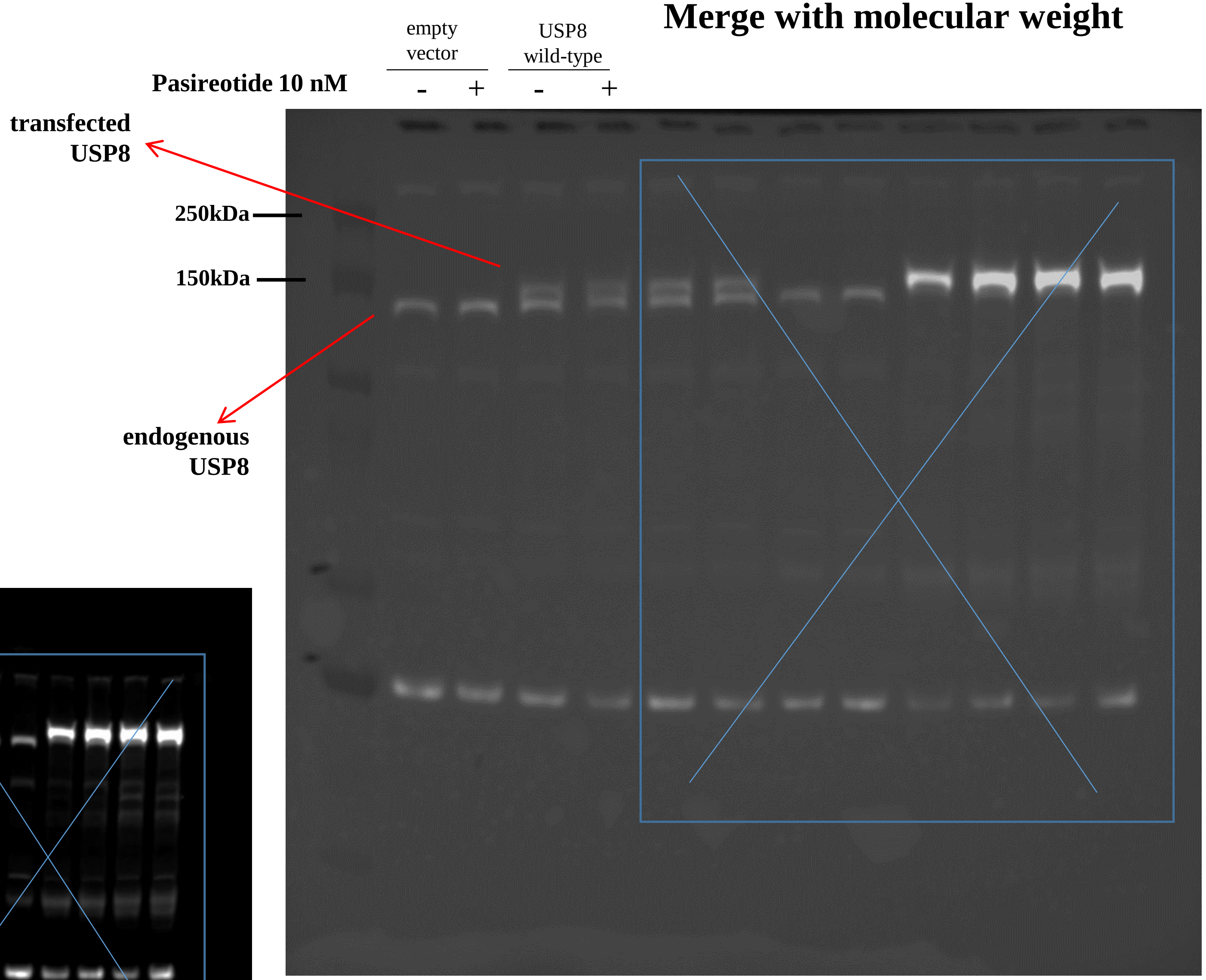

USP8
molecular weight

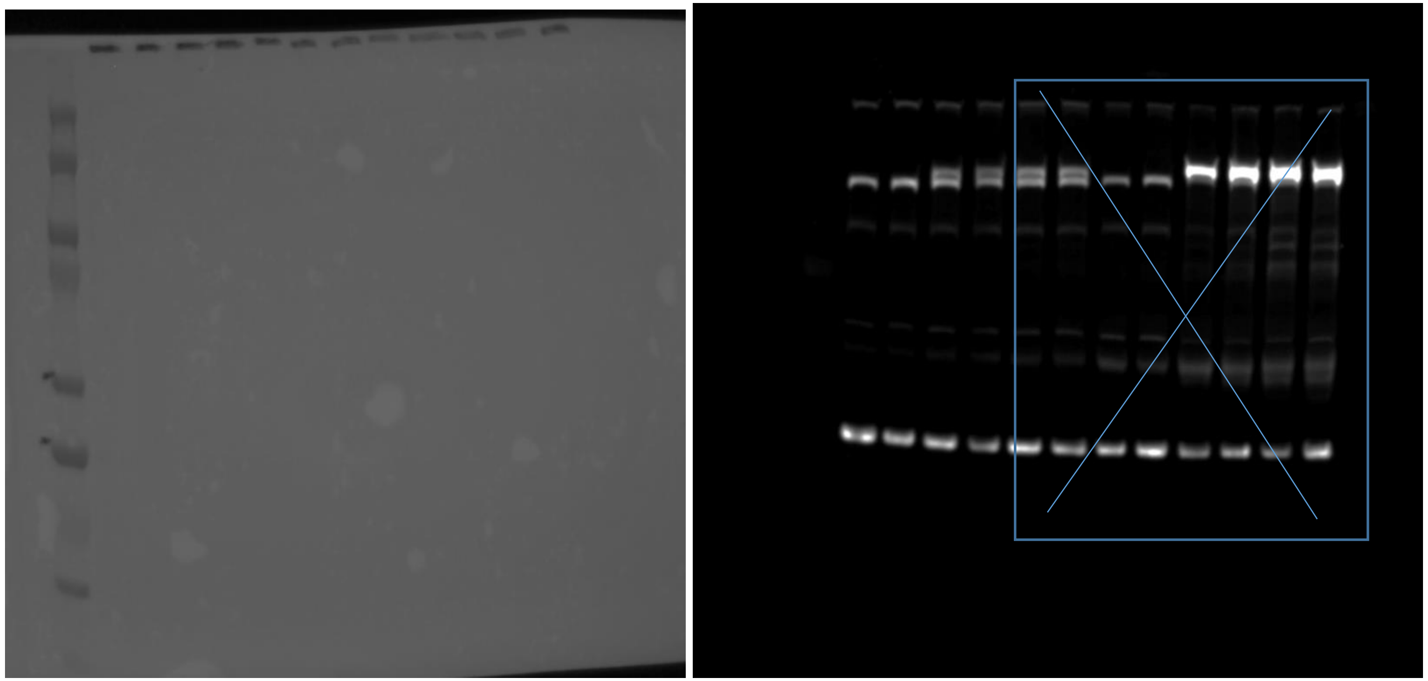

Fig. S6 b

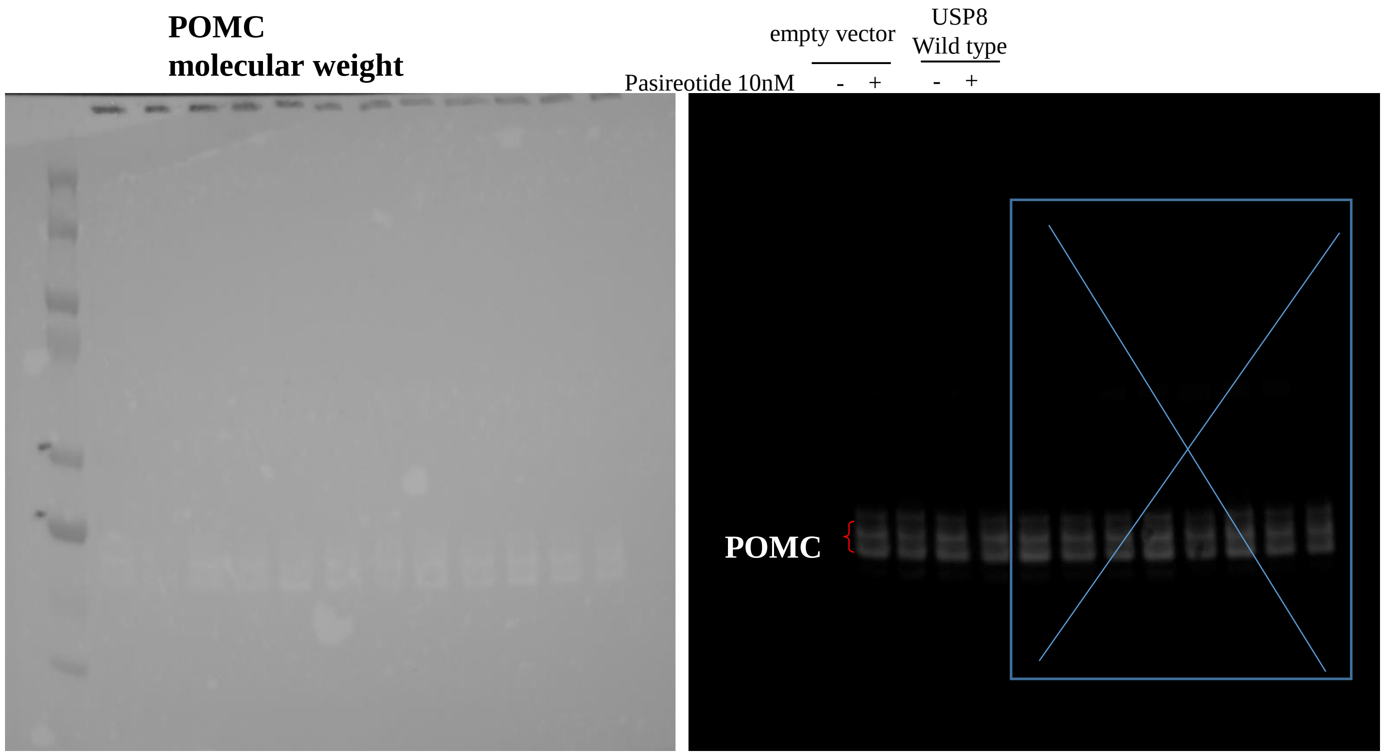

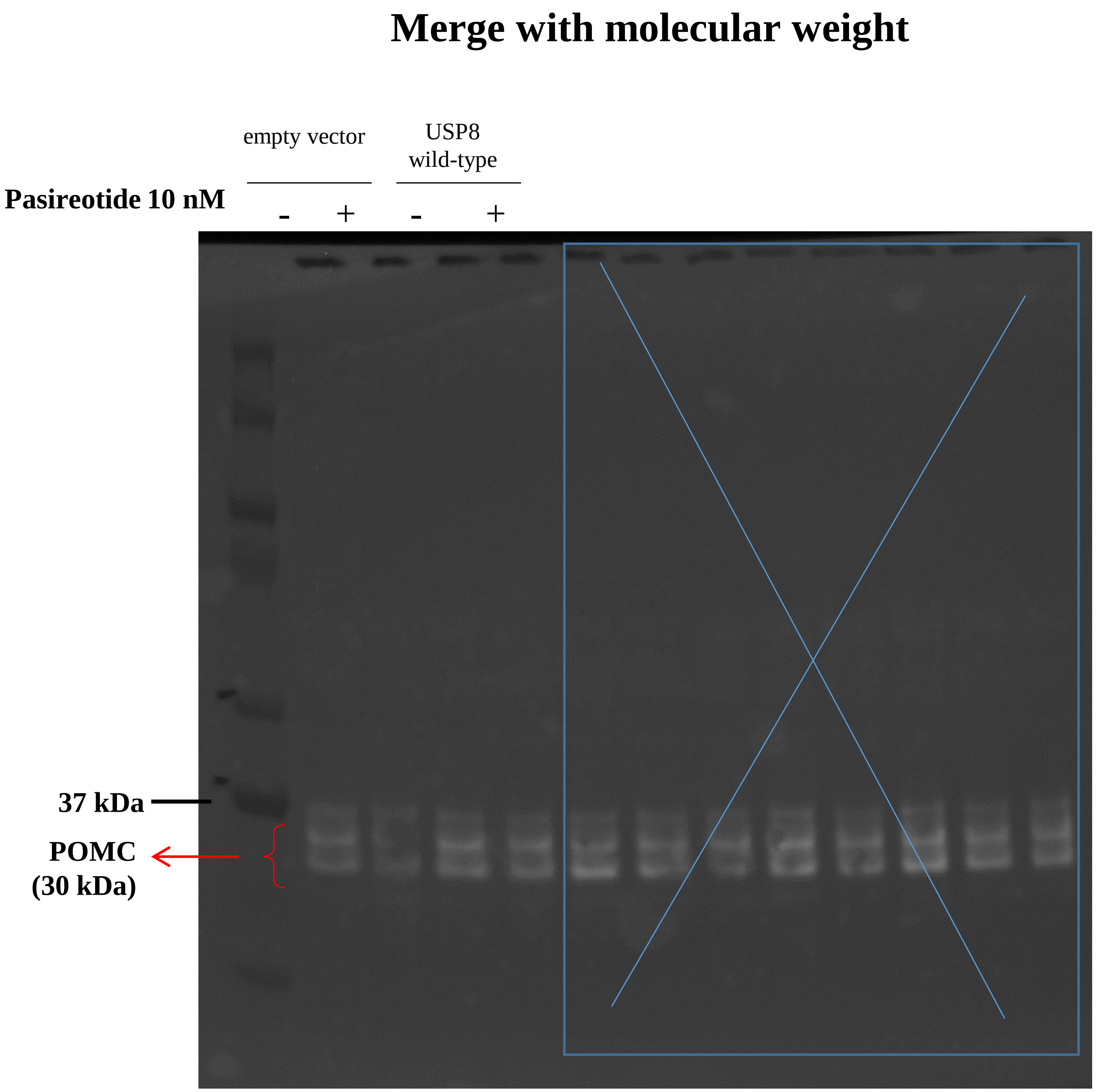

Fig. S6 b Original western blot and merge with molecular weight. Immunoblot of POMC in AtT-20 cells transfected with USP8 wild type and treated with pasireotide. Membranes were incubated with POMC antibody.

Fig. S6 c

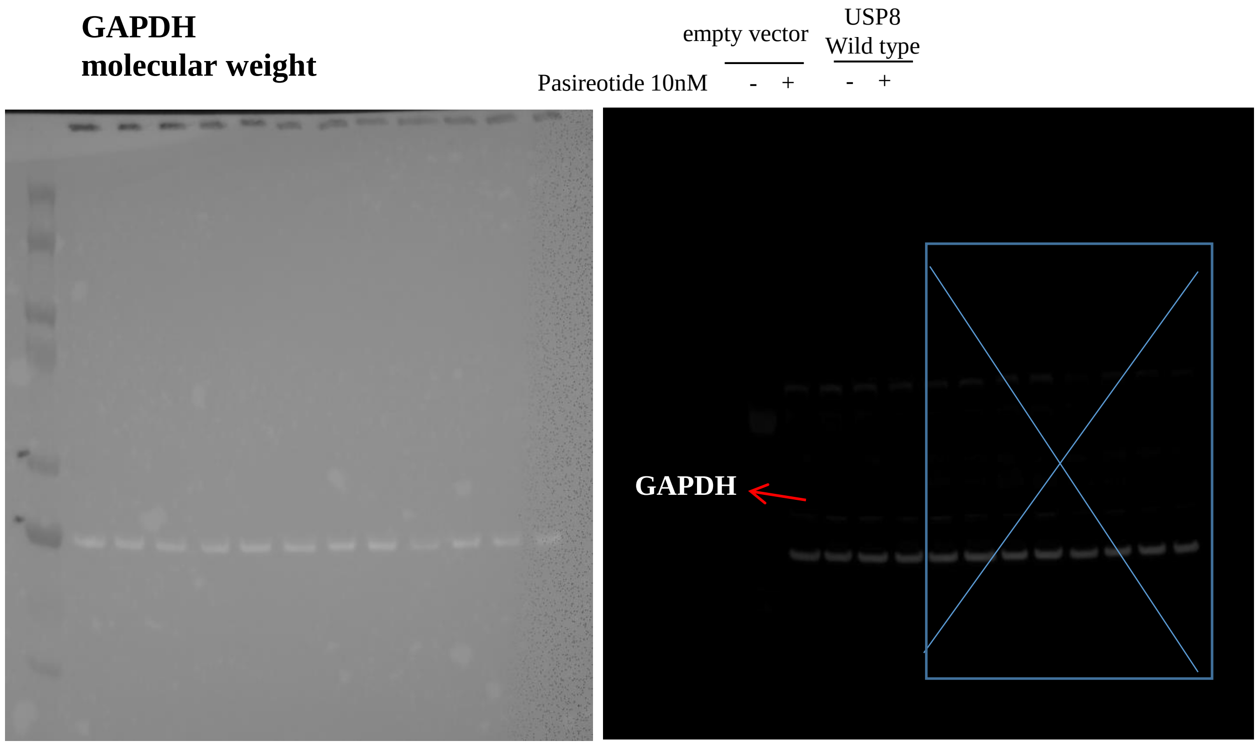

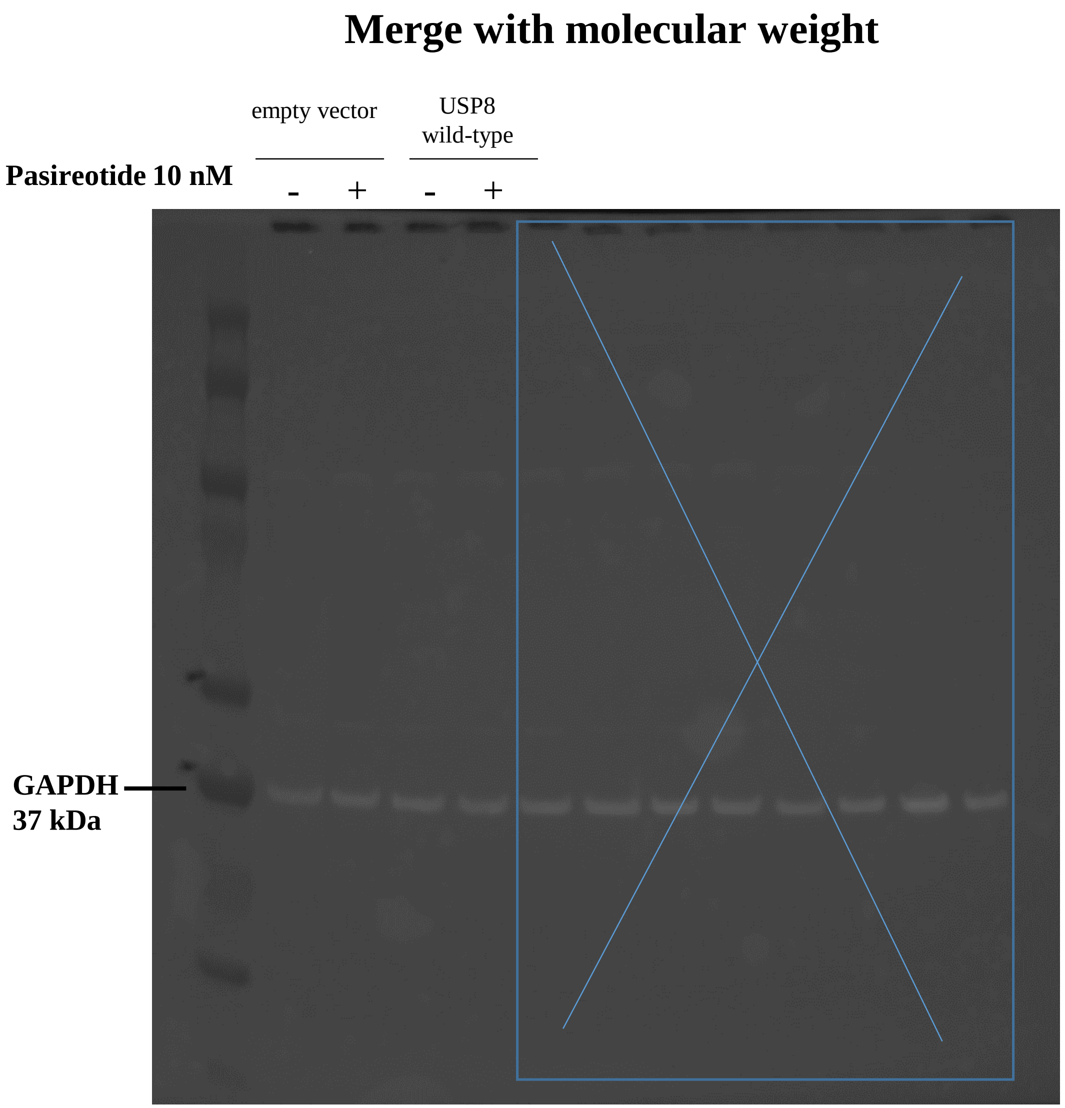

Fig. S6 c Original western blot and merge with molecular weight. Immunoblot of GAPDH in AtT-20 cells transfected with USP8 wild type and treated with pasireotide. Membranes were incubated with GAPDH antibody.
